# Effectiveness of work-related interventions for return to work in people on sick leave: a systematic review and meta-analysis of randomized controlled trials

**DOI:** 10.1186/s13643-022-02055-7

**Published:** 2022-09-05

**Authors:** Alexander Tingulstad, Jose Meneses-Echavez, Line Holtet Evensen, Maria Bjerk, Rigmor C. Berg

**Affiliations:** 1 Oslo Metropolitan University, Pilestredet 44, 0167 Oslo, Norway; 2 Division for Health Services, Norwegian Institute of Public Health, Oslo, Norway; 3 Facultad de Cultura Fisica, Deporte y Recreación, Universidad Santo Tomás, Bogotá, Colombia; 4 Department of Community Medicine, University of Tromsø, Tromsø, Norway

**Keywords:** Return to work, Vocational rehabilitation, Meta-analysis, Sick leave

## Abstract

**Background:**

Long-term sick leave is a serious concern in developed countries and the cost of sickness absence and disability benefits cause major challenges for both the individual and society as a whole. Despite an increasing body of research reported by existing systematic reviews, there is uncertainty regarding the effect on return to work of workrelated interventions for workers with different diagnoses. The objective of this systematic review was to assess and summarize available research about the effects of work-related interventions for people on long-term sick leave and those at risk of long-term sick leave.

**Methods:**

We conducted a systematic review in accordance with international guidelines. Campbell Collaboration (Area: Social Welfare), Cochrane Database of Systematic Reviews, Cochrane Central Register of Controlled Trials, Embase, Epistemonikos, MEDLINE, PsycINFO, Scopus, and Sociological Abstracts were systematically searched in March 2021. Two authors independently screened the studies. We conducted risk of bias assessments and meta-analyses of the available evidence in randomized controlled trials (RCTs). The remaining comparisons were synthesized narratively. The certainty of evidence for each outcome was assessed.

**Results:**

We included 20 RCTs comprising 5753 participants at baseline from 4 different countries. The studies had generally low risk of bias. Our certainty in the effect estimates ranged from very low to moderate. Eight different interventions were identified. Meta-analysis revealed no statistically significant difference between multidisciplinary rehabilitation (MR) and usual care (US) (Risk Ratio [RR] 1.01; Confidence Interval [CI] 95% 0.70-1.48 at 12 months follow-up) and between MR and other active intervention (Risk Ratio [RR] 1.04; Confidence Interval [CI] 95% 0.86-1.25 at 12 months follow-up). Remaining intervention groups revealed marginal, or no effect compared to the control group. The results for the secondary outcomes (self-efficacy, symptom reduction, function, cost-effectiveness) showed varied and small effects in the intervention groups.

**Conclusion:**

Overall, the present data showed no conclusive evidence of which work-related intervention is most effective for return to work. However, a handful of potential interventions exist, that may contribute to a foundation for future research. Our findings support the need for adequately powered and methodologically strong studies.

**Supplementary Information:**

The online version contains supplementary material available at 10.1186/s13643-022-02055-7.

## Background

Long-term sick leave is a serious concern in developed countries, and the cost of sickness absence and disability benefits cause major challenges for both the individual and society as a whole [1]. Thus, assisting workers back to work with determined and effective interventions is important to reduce the individual, economic, and societal burden. Furthermore, staying at work is beneficial both for people’s physical and mental health and for their personal identity and social status [2]. Compared to other developed countries, Norway has a higher proportion of people on sick leave. This might be influenced by several factors, such as high work participation and generous sick leave arrangements [3, 4]. Other countries with relatively high proportions on sick leave are Austria, Belgium, and Germany [4]. Sick leave arrangements vary across countries, with regard to the length of benefits, percentage of salary covered by the benefits, the possibility of losing a job while on sick leave, and low work participation [3]. As a result, country comparisons within the topic work and welfare are challenging.

Work-related interventions aim to facilitate return to work (RTW) for people on sick leave or help people at risk of sick leave to stay at work. Their focus is often to strengthen workers’ work-ability, emphasize work self-efficacy, and overcome obstacles for work participation. However, work-related interventions differ widely in content, duration, and components. Examples include social worker interventions, health-related interventions such as physiotherapy and psychological therapy, workplace interventions, occupational therapy, in- or outpatient rehabilitation, and work or health education [5]. Unfortunately, studies have struggled to show positive long-term effects of the interventions in terms of RTW or sick leave status [5, 6]. While the reasons for this likely are multifactorial, a recent study reported that the management of the RTW process can be experienced as challenging for caseworkers due to the psychosocial factors involved, etiology of the illness, and that the capabilities of the workers are intertwined and complicated to disentangle [7]. Furthermore, for people on sick leave with a musculoskeletal disorder, there might be a mismatch between the mostly biomedical legislation for the sick leave status and the biopsychosocial requirements and challenges for the workers [7]. Some studies have shown that patients with different diagnoses and pain regions share many of the same prognostic factors for RTW and barriers of RTW [8–10]. These similarities among people on sick leave or at risk of sick leave argue for including people with various diagnoses and pain regions in studies conducted within work and welfare.

Despite an increasing body of research reported by existing systematic reviews, there is uncertainty regarding the effect on RTW of work-related interventions for workers with different diagnoses [5, 6]. A systematic review assessing the effects of work-related interventions without restrictions in type of interventions and diagnoses might contribute to greater certainty regarding the effect among policymakers and experts in the field.

This systematic review aims to identify, assess, and summarize available research about the effects of work-related interventions for people on long-term sick leave and those at risk of long-term sick leave.

## Methods

### Protocol and registration

The protocol for this systematic review is registered in Norwegian [11] and English [12] (Appendix 1). The review is based on and is an update of a report from 2021, published in Norwegian [13]. We report in accordance with the Preferred Reporting Items for Systematic Reviews and Meta-Analyses (PRISMA) [14].

### Eligibility criteria

#### Study design

We included randomized controlled trials (RCTs).

#### Population

We included studies recruiting employees with partial (≥50%) or full sick leave for a period of 1 to 24 months, irrespective of the reason for sick leave and the setting of benefit scheme. Studies could include employees on shorter sick leave, who were at risk of long-term sick leave or unemployed as long as the proportion of such employees was <30% in any study. Participants could be at risk of long-term sick leave for any reason, but they had to be characterized as at-risk by the researchers conducting the RCT. We enforced no age restrictions for participants.

#### Experimental intervention

The intervention being assessed had to be a RTW intervention where the active health component accounted for ≥70% of the intervention and the objective of the intervention was to promote RTW.

We excluded workplace interventions without a health component and individual placement and support and supported employment, because the effect of these interventions was recently assessed in another systematic review conducted by the Norwegian Institute of Public Health (NIPH) [15].

#### Control intervention

Usual care (UC) by conventional case management and healthcare providers or any active intervention.

#### Outcome measures

##### Primary outcome

To be included, a study had to provide data for an outcome related to sickness absence or RTW, including time to return to work, cumulative sick leave, proportion of participants at work, or time at work before the new period of sick leave. That is, sickness absence or RTW could be measured and reported as the number of days of sick leave until RTW, total number of days of (partial/full) sick leave during follow-up, rate of RTW at follow-up, or similar.

##### Secondary outcomes

We included self-efficacy and work motivation as measured by a psychometric scale such as Work Self-Efficacy Scale [16], Return-to-Work Self-efficacy Questionnaire [17], and Motivation at Work Scale [18]. We also included symptom reduction and physical/social/cognitive function as measured by scales such as the Short Form 36 Health Survey [19], EuroQol 5 Dimension [20], the Four-Dimensional Symptom Questionnaire [21], and Work Limitations Questionnaire [22]. Lastly, we included cost-effectiveness.

#### Other

Only studies published in the year 2000 or later, in order to ensure relevance for current national approaches. The publications could be in any language that the researchers or close colleagues mastered, including Danish, English, Finnish, French, German, Italian, Norwegian, Portuguese, Spanish, and Swedish. Only studies reported in full text were eligible, because a full text is necessary to conduct the risk of bias of studies, ascertain the PICO (population, intervention, comparison, outcome) elements, and retrieve all necessary data [23].

### Data sources and searches

A research librarian at the NIPH developed a systematic search strategy, which was peer-reviewed by another information specialist at the NIPH. The search was conducted in the following electronic databases: Campbell Collaboration (Subject area: Social Welfare), Cochrane Database of Systematic Reviews (CDSR), Cochrane Central Register of Controlled Trials (CENTRAL), Embase, Epistemonikos (Broad Synthesis & Systematic Reviews), MEDLINE, PsycINFO, Scopus, and Sociological Abstracts (including Social Services Abstracts). The final search strategy is available in Appendix 2. Searches of gray literature were conducted in Google Scholar and webpages of relevant Scandinavian institutions. Additionally, one reviewer screened the reference lists of included studies and relevant systematic reviews. The search was conducted in May 2020 and updated in March 2021.

### Data collection and analysis

#### Selection of studies

We imported the search records to EndNote and deleted duplicates [24]. The search records were then imported to EPPI-Reviewer [25] and identified titles and abstracts were independently screened by two researchers. Relevant references were obtained in full text and independently assessed by two researchers. At both screening levels, an inclusion/exclusion form guided the selection process, and disagreements were solved by discussion or with a third researcher.

#### Data extraction and management

A standardized form was used to extract the following data from each included study: Study aim, study design, country, description of the population including diagnosis and sick leave duration, description of the intervention and controls, outcomes, follow-up period, source of sick leave information, outcome results. Data was extracted by one researcher and cross-checked by another researcher. Disagreements were resolved between the researchers or by the involvement of a third researcher if necessary. To ensure thorough and consistent descriptions of the interventions, we used the template for intervention description and replication (TIDieR) checklist and guide [26].

#### Risk of bias assessment

We used the Cochrane risk of bias tool, which includes the domains sequence generation (whether the study used a randomized sequence of assignments), allocation concealment (whether the study had adequate concealment of the allocation sequence from those involved in the enrolment and assignment of participants), blinding (whether participants and providers were masked to participants’ assignment), incomplete outcome data (whether there was missing outcome data that raise the possibility that the observed effect estimate is biased), selective outcome reporting (whether there was selection of a subset of the original variables recorded, on the basis of the results), and other sources of bias. Each domain in the tool comprises a judgment in a ‘Risk of bias’ traffic-light table. The judgment for each domain entry for each study involves assessing the risk of bias as “low risk,” as “high risk,” or as “unclear risk,” with the last category indicating either lack of information or uncertainty over the potential for bias [23]. Two independent researchers conducted the assessments and when disagreements occurred, they reached a consensus for a final decision with discussion.

#### Data synthesis

We summarized descriptive data of the included studies, such as year of publication, country, age of participants, characteristics of the intervention and control, and outcome measures. With regard to results, outcome data for the primary outcome was entered into Review Manager version 5, which is a software developed by Cochrane for preparing reviews, performing meta-analyses, and presenting the results graphically [27]. Dichotomous outcomes were expressed as risk ratios (RRs) and 95% confidence intervals (CIs). We analyzed continuous outcomes using the mean difference (MD) with 95% CI or standardized mean difference (SMD), if the outcomes had different units or scales of measurement. We performed meta-analyses when the studies had the same outcomes and were sufficiently similar in terms of population, intervention, comparison, and effect measurements, using random effects models. We examined between-study heterogeneity using visual inspection of CIs, the Chi-square test, and *I*-square statistic, quantifying the degree of heterogeneity as follows: 0% to 40% might not be important; 30% to 60% may represent moderate heterogeneity; 50% to 90% may represent substantial heterogeneity; 75% to 100% considerable heterogeneity [23]. When possible, we explored reasons for heterogeneity. Judgments regarding meta-analyses were based on recommendations in the Cochrane Handbook, which states that studies need to be sufficiently homogeneous in terms of participants involved, interventions, and outcomes to provide a meaningful summary [23]. When studies were too heterogeneous to justify meta-analyses and or data were missing, we synthesized results narratively in text and tables in accordance with the Synthesis Without Meta-analysis (SWIM) published recommendations [28]. In the analyses, we considered subgroups of different populations and interventions.

#### Certainty of evidence

We used the GRADE approach (Grading of Recommendations Assessment, Development, and Evaluation) to assess and report the certainty of the evidence for each primary outcome [29]. Reasons for downgrading the certainty in evidence are study limitations, inconsistency between studies, indirectness of evidence, imprecision, and reporting bias. We used the GRADE standard definitions and phrases when communicating the certainty of the evidence. The certainty for the documentation was assessed by two researchers and was summarized for each comparison in the summary of findings tables.

## Results

### Results of the literature search and study selection

**Fig. 1:**
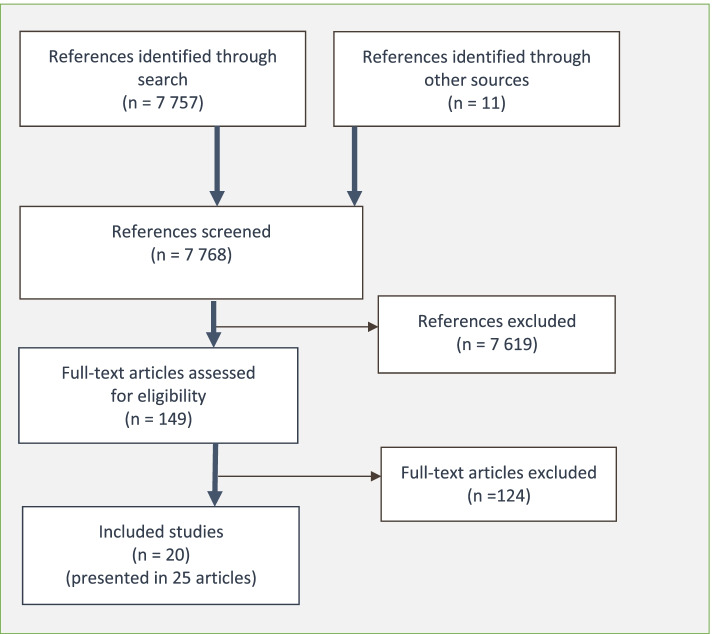
Flow diagram summarizing the search process and the screening

We identified 7768 references. After screening the titles and abstracts, we assessed 164 full-text articles. We included 20 studies, which were presented in 25 articles (Fig. 1). Excluded studies after full-text assessment are presented in Appendix 3. No eligible studies were excluded based on not being available in full text.

### Characteristics of included studies

Table 1 shows the characteristics of our included studies [30–54]. The studies were published between 2001 and 2020 and were conducted in Denmark (*n*=7), the Netherlands (*n*=6), Norway (*n*=4), and Sweden (*n*=3). In total, the 20 studies included 5753 male and female participants (range 34–1757), who had a mean age of 41 (range 16–65).

**Table 1:** Characteristics of the included studies (*N*=20 studies (presented in 25 articles))

Study (country)	Population	Intervention	Comparison	Outcomes
Aasdahl et al., 2018 [30] (Norway)	*N*=168, 45 (18–60) yrs, 77% women, CMDs, MSDs, median sick leave 224 days	Multi-disciplinary rehabilitation (12 mo)	ACT	Sickness absence days RTW 4 weeks
Gismervik et al., 2018 [31] (Norway)	*N*=166, 46 (18–60) yrs, 81% women, CMDs, MSDs, mean sick leave 210 days	Multi-disciplinary rehabilitation (12 mo)	ACT	Sickness absence days RTW 4 weeks Pain Anxiety Depression Subjective health complaints HRQoL
Brendbekken et al., 2017 [32] (Norway)	*N*=284, 40 (20–60) yrs, 55% women, MSDs, mean sick leave 147 days	Multi-disciplinary intervention (24 mo)	Brief intervention	Full and partly RTW
Brouwers et al., 2006 [33] (the Netherlands)	*N*=194, 40 (18–60) yrs, 58% women, MSDs, <3 mo sick leave	Problem-solving approach (18 mo)	UC	Days of sick leave Anxiety Depression
Bültmann et al., 2009 [34] (Denmark)	*N*=113, 44 yrs, 55% women, MSDs, mean sick leave 39 days	Multi-disciplinary rehabilitation (12 mo)	UC	Hours of sickness absence Work status Pain intensity Functional disability
Dalgaard et al., 2017 [35] (Denmark)	*N*=163, 45 (28–60) yrs, 74% women, adjustment disorder, stress reaction, mild depression, <4 mo sick leave	W-CBT (10 mo)	(1) Clinical examination and UC (2) UC	Time until lasting RTW Sick leave status Length of sick leave
De Weerd et al., 2016 [36] (the Netherlands)	*N*=60, 40 yrs, 58% women, CMDs, mean sick leave 42 days	W-CBT with additional dialogue meeting	W-CBT	Time to first RTW Time to full RTW Mental health
Hees et al., 2013 [37] (the Netherlands)	*N*=117, 43 (18–65) yrs, 47% women, depression, >8 weeks sick leave	Adjuvant occupational therapy (18 mo)	UC	Absenteeism Time until partial/full RTW Depression Health-related function At-work functioning
Jensen et al., 2011, 2012 [38, 39] (Denmark)	*N*=351, 42 (16–60) yrs, 54% women, low back pain, 3–16 weeks sick leave	Multi-disciplinary rehabilitation and brief intervention (24 mo)	Brief intervention	RTW 4 weeks Pain Disability Fear-avoidance Quality of life
Lambeek et al., 2010, 2010 [40, 41] (the Netherlands)	*N*=134, 46 (18–65) yrs, 44% women, low back pain, median sick leave 142 days	Multi-disciplinary rehabilitation (12 mo)	UC	RTW 4 weeks Pain intensity Functional status HRQOL Cost-effectiveness
Lindell et al., 2008 [42] (Sweden)	*N*=123, 42 (<59) yrs, 34% women, back and neck pain, 42–730 days sick leave	W-CBT (18 mo)	UC	RTW for 30 days RTW chance Days of sick leave
Marhold et al., 2001 [43] (Sweden)	*N*=72, 46 (25–60) yrs, 100% women, MSDs, mean sick leave 3 mo	W-CBT (6 mo)	UC	Days on sick leave Pain Coping Disability Depression
Martin et al., 2013 [44] (Denmark)	*N*=196, 41 (20–60) yrs, 69% women, CMDs, mean sick leave 8 weeks	Multi-disciplinary rehabilitation (12 mo)	UC	Time to RTW Work status
Meijer et al., 2006 [45] (the Netherlands)	*N*=34, 39 (18–65) yrs, 61% women, MSDs, 4–20 weeks sick leave	Multi-disciplinary rehabilitation (12 mo)	UC	RTW Pain Disability Physical function Cost-effectiveness
Moll et al., 2018 [46] (Denmark)	*N*=168, 40 (18–60) yrs, 69% women, neck and shoulder pain, 4–12 weeks sick leave	Multi-disciplinary rehabilitation (12 mo)	Brief multi-disciplinary intervention	RTW 4 weeks Pain Disability
Poulsen et al., 2014 [47] (Denmark)	*N*=1757, 41 yrs, 59% women, CMDs and somatic disorders, ≥8 weeks sick leave	Multi-disciplinary rehabilitation (12 mo)	UC	RTW 1 week
Momsen et al., 2016 [48] (Denmark)	*N*=443, 44 (18–65) yrs, 62% women, MSDs and CMDs	Multi-disciplinary rehabilitation (12 mo)	UC	RTW 4 weeks
Myhre et al., 2014 [49] (Norway)	*N*=405, 40 (18–60) yrs, 44% women, back and neck pain, 4–12 weeks sick leave	Work-focused multi-disciplinary rehabilitation (12 mo)	Multi-disciplinary intervention and brief multi-disciplinary intervention	RTW 5 weeks
Netterström et al., 2013 [50] (Denmark)	*N*=199, 44 yrs, 82% women, work-related stress, mean sick leave 70 days	Stress treatment program (3 mo)	(1) UC with psychologist (2) Wait list	RTW Psychological symptoms Workability Degree of stress
Salomonsson et al., 2017, 2020 [51, 52] (Sweden)	*N*=211, 42 (21–64) yrs, 79% women, CMDs, 4–26 weeks sick leave	Work-focused CBT (12 mo)	(1) CBT (2) Combination treatment	Days of sick leave Psychiatric symptoms QoL Workability
Skagseth et al., 2019 [53] (Norway)	*N*=175, 45 (18–60) yrs, 77% women, CMDs, MSDs, mean sick leave 184 days	Multi-disciplinary rehabilitation with additional workplace intervention (12 mo)	Multi-disciplinary rehabilitation	Days of sickness absence Time until sustainable RTW 4 weeks
Volker et al., 2015 [54] (the Netherlands)	*N*=220, 44 yrs, 59% women, CMDs, median sick leave 70 days	E-health module with collaborative occupational health care (12 mo)	UC	Days of sickness absence until first RTW Days of sickness absence until full RTW Total number of days of sickness absence Depression Anxiety Remission of psychological symptoms

*Legend*: *ACT* acceptance and commitment therapy, *CBT* cognitive behavioral therapy, *CMD* common mental disorders, *mo* months, *MSD* musculoskeletal disorder, *HRQoL* health-related quality of life, *UC* usual care, *yrs* years

Time on sick leave at baseline ranged from 5 to 52 weeks. The studies investigated both generic and specific disorders as a reason for sick leave. Four studies included people with common mental disorders (CMD), four studies focused on both CMD and musculoskeletal disorders, and four studies concerned generic musculoskeletal disorders. The remaining eight studies assessed specific diagnoses: back- and neck pain (*n*=2), low back pain (=2), depression, adjustment disorders and depression, neck and shoulder pain, and stress.

#### Interventions and control conditions

There were eight different interventions, which had a duration from three weeks to eight months. Ten studies assessed multidisciplinary rehabilitation (MR) in different varieties, such as inpatient- or outpatient clinics, day visits or overnight stays [30, 32, 34, 38, 41, 44–47, 49]. Work-focused cognitive behavioral therapy (W-CBT) was assessed by four studies [35, 42, 43, 51], and the remaining six interventions, investigated by one study each, were occupational therapy [37], activating problem-solving approach [33], stress reduction program [50], additional dialogue meeting [36], additional workplace intervention [53] and an electronic health module [54]. In addition to being diverse with regard to content and duration, a variety of professions were involved in the interventions, including physicians, physiotherapists, social workers, psychologists, and occupational therapists.

The control conditions were either usual care or other active intervention. The content of usual care, which was applied in 11 studies, was diverse and varied according to national standards. Nine studies compared the experimental intervention to usual care and or to another active intervention.

#### Risk of bias

**Fig. 2:**
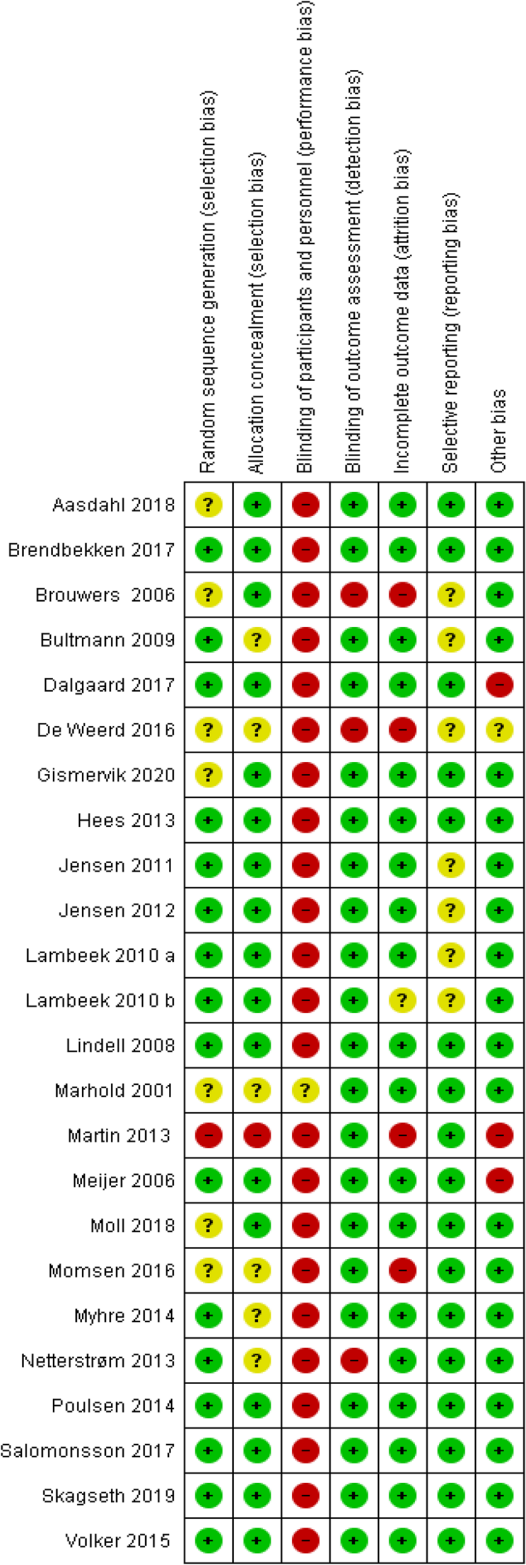
Risk of bias in included studies. The traffic light plot presents the domain level judgments for each study: green (+) means no bias, yellow (?) means unclear bias, red (−) means high risk of bias

Our risk of bias assessment of the included studies is summarized and presented in Fig. 2. The studies had generally low risk of bias, especially regarding selection bias, attrition bias, and other biases. However, in all but one study, there was no blinding of participants and personnel, thus they were assessed as having a high risk of bias on blinding. This is because blinding of both participants and personnel is preferred to avoid performance bias [23].

### Outcome results

#### Primary outcomes

##### Return to work

As per our inclusion criteria, all studies operationalized RTW either as a time to RTW or time to sustainable RTW, days on sick leave, proportion of participants at work, sick leave, or work status. The effect estimates of work-related outcomes are presented in Table 2. Due to the low number of comparable studies, we could not conduct subgroup analyses. Our GRADE assessments are presented in Table 3 and show that our certainty in the effect estimates ranged from very low to moderate for the primary outcome.

**Table 2:** Effect estimates of work-related outcomes

Study	Intervention/comparison	Outcome (follow-up)	Result/effect estimate (95% CI)
Return to work			
Bültmann et al., 2009 [34]	MR vs. UC	Days of sick leave (18 mo)	RR = 1.24 (0.97–1.59)
Lambeek et al., 2010, 2010 [40, 41]	MR vs. UC	Days of sick leave until full sustainable RTW (12 mo)	82 days (IQR 51–164 days) vs. 175 days (91–365)
Martin et al., 2013 [44]	MR vs. UC	Time until RTW (12 mo)	RR = 0.74 (0.60–0.91)
Meijer et al., 2006 [45]	MR vs. UC	RTW (12 mo)	RR = 1.19 (0.81–1.74)
Momsen et al., 2016/ Poulsen et al., 2014 [47, 48]	MR vs. UC	RTW for 4 weeks (12 mo) RTW for 1 week (12 mo)	RR = 0.92 (0.78–1.08) HR = 1.12 (0.97–1.29) and HR = 0.80 (0.63–1.03)
Aasdahl et al., 2018/ Gismervik et al., 2020 [30, 31]	MR vs. acceptance and commitment therapy	Sickness absence days Time until sustainable RTW for 1 month (12 mo)	RR = 0.86 (0.65–1.15) RR = 1.50 (1.08–2.08)
Brendbekken et al., 2017 [32]	MR vs. brief intervention	Full and partly RTW (12 mo) Full and partly RTW (24 mo)	RR = 0.92 (0.71–1.20) RR = 0.94 (0.73–1.21)
Jensen et al., 2011, 2012 [38, 39]	MR vs. brief clinical intervention	Sustainable RTW for 4 weeks (24 mo)	RR = 0.93 (0.82–1.06)
Moll et al., 2018 [46]	MR vs. brief intervention	Sustainable RTW for 4 weeks (12 mo)	RR = 1.04 (0.81–1.34)
Myhre et al., 2014 [49]	Work-focused MR vs. MR and brief intervention	RTW for 5 weeks (12 mo)	RR = 0.93 (0.82–1.05)
Dalgaard et al., 2017 [35]	W-CBT vs. UC	Time until lasting RTW (10 mo)	HR = 1.57 (1.01–2.44)
Lindell et al., 2008 [42]	W-CBT vs. UC	RTW for 30 days (18 mo)	57% vs. 57%
Marhold et al., 2001 [43]	W-CBT vs. UC	Days of sick leave (4 mo) Days of sick leave (6 mo)	25.4 days vs. 37.2 days 21.0 days vs. 39.7 days
Salomonsson et al., 2017, 2020 [51, 52]	W-CBT vs. CBT or combination treatment	Days of sick leave (12 mo)	27 (− 8.7–62.8) days less sick leave; 18 (−15.8–52.1) days less sick leave
Brouwers et al., 2006 [33]	Problem-solving approach vs. UC	Days of sick leave (18 mo)	106 (SD 0.87) days in the intervention group vs. 121 (SD 0.94) days in the control group
De Weerd et al., 2016 [36]	W-CBT with additional dialogue meeting vs. W-CBT	Time to first RTW Time to full RTW	MD = −2.02 (−28.2, 24.2) MD = 48 (−2.9, 100.8)
Hees et al., 2013 [37]	Adjuvant occupational therapy vs. UC	Time until partial RTW (18 mo) Time until full RTW (18 mo)	HR = 0.72 (0.44–1.11) HR = 0.93 (0.57–1.53)
Netterström et al., 2013 [50]	Stress treatment program vs. UC with psychologist or waiting list	RTW (3 mo)	OR = 8.1 (3.2–20.7)
Skagseth et al., 2019 [53]	MR with additional workplace intervention vs. MR	Days of sickness absence (12 mo) Time until sustainable RTW for 4 weeks (12 mo)	130 (IQR 81–212) days in the intervention group vs. 115 (IQR 53–183) days in the control group HR = 0.74 (0.48–1.6)
Volker et al., 2015 [54]	E-health module with collaborative occupational health care vs. UC	Days of sickness absence until first RTW (12 mo) Days of sickness absence until full RTW (12 mo) Total number of days of sickness absence (12 mo)	HR = 1.39 (1.04–1.87) HR = 1.29 (0.91–1.81) 174 (IQR 100.0–321.0) days vs. 228 (IQR 111.0–365.0) days

*Legend*: *CBT* cognitive behavioral therapy, *CMD* common mental disorders, *HR* hazard ratio, *IQR* interquartile range, *MD* mean difference; *mo*, months, *MR* multidisciplinary rehabilitation, *MSD* musculoskeletal disorders, *HRQoL* health-related quality of life, *OR* odds ratio, *RR* risk ratio, *RTW* return to work, *UC* usual care, *W-CBT* work-related cognitive behavioral therapy

**Table 3:** Certainty of evidence of work-related interventions

Population: Adults on full or partly sick leave Countries: Denmark, Norway, Sweden, The Netherlands Intervention: Work-related interventions Comparison: Usual care or other active intervention						
Outcome, follow-up time	Relative effect (95% KI)	Anticipated absolute effects (95% CI)			Number of participants (studies)	Quality of evidence (GRADE)
		Assumed risk with control	Assumed risk with intervention	Absolute difference (intervention minus control)		
Multidisciplinary rehabilitation vs. UC						
Return to work (12 mo)	RR = 1.01 (0.70–1.48)	72 per 100	73 per 100 (50 to 107)	1 more person	321 participants (3 RCTs)	⨁◯◯◯ Very low^a,b,c^
Return to work (12 mo)	-	-	-	One study found no difference between the groups. One study found that the intervention group had shorter time on sick leave than the control group.	1891 participants (2 RCTs)	⨁◯◯◯ Very low^a,b,c^
Multidisciplinary rehabilitation vs. active treatment						
Return to work (12 mo)	RR = 1.04 (0.86–1.25)	59 per 100	61 per 100 (51 to 74)	Three more persons (14 less to 25 more)	851 participants (4 RCTs)	⨁◯◯◯ Very low^a,b^
Return to work (24 mo)	RR = 0.94 (0.84–1.05)	63 per 100	59 per 100	Four less persons (16 less to 5 more)	635 participants (2 RCTs)	⨁⨁⨁◯ Moderate^d^
W-CBT vs. UC						
Return to work	-	-	-	Two studies showed faster RTW in the intervention group compared to UC at 4 and 6 mo. One study showed no difference between the groups at 18 mo.	311 participants (3 RCTs)	⨁⨁◯◯ Low^a^
W-CBT vs. active intervention						
Return to work (12 mo)	27 (− 8.7, 62.8) days less sick leave than control group 1, 18 (− 15.8, 52.1) days less sick leave than control group 2	-	-	No difference between the groups in number of sick days	211 participants (1 RCTs)	⨁◯◯◯ Very low ^e,f^
Problem-solving approach vs. UC						
Return to work (3 mo)	-	39 per 100	37 per 100	No difference between the groups in days on sick leave	194 participants (1 RCT)	⨁◯◯◯ Very low^g,h^
Return to work (6 mo)	-	62 per 100	58 per 100	No difference between the groups in days on sick leave	194 participants (1 RCT)	⨁◯◯◯ Very low^g,h^
Return to work (18 mo)	HR = 1.04 (0.76–1.42)	79 per 100	85 per 100	No difference between the groups in days on sick leave	194 participants (1 RCT)	⨁◯◯◯ Very low^g,h^
Additional dialogue meeting vs. active treatment						
Return to work	MD = 48 (− 2.9, 100.8)			No difference between the groups in days until full RTW	60 participants (1 RCT)	⨁◯◯◯ Very low^f,i^
Days to first RTW	MD = − 2.02 (− 28.2, 24.2)			No difference between the groups in days until the first RTW	60 participants (1 RCT)	⨁◯◯◯ Very low^f,i^
Adjuvant occupational therapy vs. UC						
Full return to work (18 mo)	HR = 0.93 (0.57–1.53)	-	-	No difference between the groups	117 participants (1 RCT)	⨁◯◯◯ Very low^a,f^
Partly return to work (18 mo)	HR = 0.72 (0.44–1.11)	-	-	No difference between the groups	117 participants (1 RCT)	⨁◯◯◯ Very low^a,f^
Stress treatment program vs. UC or waiting list						
Return to work (3 mo)	OR = 8.1 (3.2–20.7)	-	-			⨁◯◯◯ Very low^f,i^
MR with additional workplace meeting vs. MR						
Return to work (12 mo)	HR = 0.74 (0.48–1.16)	52 per 100	42 per 100	Sustainable RTW 42% in the intervention group vs. 52% in the control group	175 participants (1 RCT)	⨁◯◯◯ Very low^d,f^
E-health module with collaborative occupational health care vs. UC						
Full return to work (12 mo)	-	Median days 178 (IQR 72.0–243.3)	Median days 131 (IQR 68.5–198)	Median of 47 days faster full RTW in the intervention group compared to the control group	131 participants (1 RCT)	⨁◯◯◯ Very low^d,f^
First return to work (12 mo)	HR = 1.39 (1.03–1.87)	77 days until the first RTW	50 days until the first RTW	27 days faster first RTW in the intervention group compared to the control group	131 participants (1 RCT)	⨁◯◯◯ Very low^d,f^

*CI* confidence interval, *RCT* randomized controlled trial, *HR* hazard ratio, *MD* mean difference, *OR* odds ratio, *RR* risk ratio, *RTW* return to work

^a^Downgraded 2 levels for risk of bias (selection and performance bias)

^b^Downgraded 1 level for inconsistency

^c^Downgraded 1 level for imprecision (confidence interval encloses negative and positive effect)

^d^Downgraded 1 level for risk of bias (performance bias)

^e^Downgraded 1 level for risk of bias (reporting and performance bias)

^f^Downgraded 2 levels for imprecision (confidence interval encloses negative and positive effect and low number of events)

^g^Downgraded 2 levels for risk of bias (selection, reporting, and other bias)

^h^Downgraded 1 level for imprecision (low number of events)

^i^Downgraded 2 levels for risk of bias (selection, performance, and attrition bias)

**Fig. 3:**
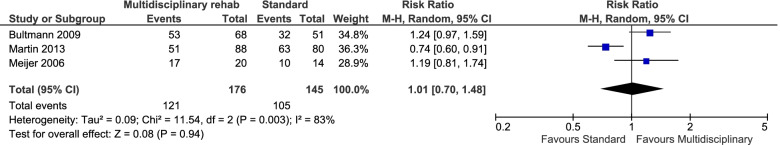
Meta-analysis of multidisciplinary rehabilitation vs. standard/usual care on RTW at 12 months follow-up

The comparison of MR versus UC included five studies, of which three could be combined in a meta-analysis (Fig. 3). There was no statistically significant difference between the groups on RTW (RR = 1.01, 95% CI = 0.70–1.48) and there was high heterogeneity. The two studies in the narrative synthesis gave varied results. We have very low certainty in the effect estimate.

**Fig. 4:**
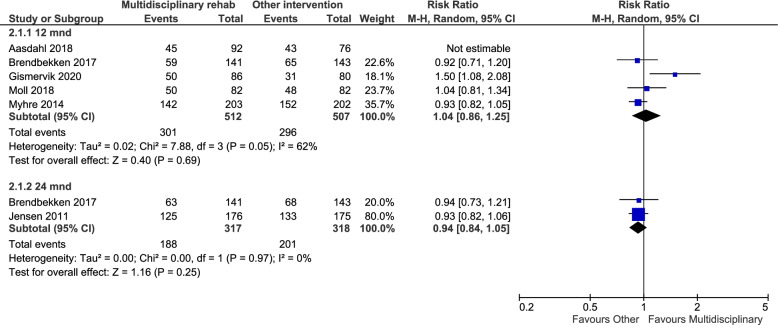
Meta-analyses of multidisciplinary rehabilitation vs. another active intervention on RTW at 12 months and 24 months follow-up

The result for the MR versus active intervention on RTW is presented in Fig. 4. Five studies could be included in the meta-analysis for 12 months of follow-up, resulting in a non-significant value of RR = 1.04, 95% CI = 0.86–1.25 (very low certainty). At 24 months follow-up the RR was 0.94, 95% CI = 0.84–1.05 (moderate certainty).

W-CBT was compared with UC in three studies, which could not be assessed in a meta-analysis due to heterogeneity. Two studies found a significant effect of W-CBT compared to UC [35, 43], while one study found no significant difference between the groups at 18 months of follow-up [42] (low certainty). One study found no significant difference between the W-CBT group and the two control groups, which received cognitive behavioral therapy (CBT) and a combination of W-CBT and CBT [51, 52] (very low certainty).

One study compared adjuvant occupational therapy to UC for participants on sick leave due to CMDs [37]. The study found no significant group differences in time until partial or full RTW at 18 months of follow-up (very low certainty).

Activating problem-solving approach versus UC was assessed by one study in a Dutch population with CMDs [33]. The study detected no significant difference between the groups in RTW (very low certainty).

One study compared a multidisciplinary stress treatment program with two groups within usual care, one with sessions with a psychologist, and one where participants were on a waiting list for treatment [50]. There was a significant difference between the groups in RTW in favor of the intervention group (very low certainty).

One study assessed the effect of an additional dialogue meeting beside W-CBT [36]. The study found no significant differences between the groups (very low certainty).

MR with additional workplace intervention versus only MR was assessed in one study [53]. The number of sick days was significantly higher in the intervention group with additional workplace intervention after 12 months of follow-up (130 days vs. 115 days) (very low certainty).

One study compared an electronic health module intervention with UC [54], and found a significant effect on time to first RTW (HR = 1.39, 95% CI = 1.03–1.87) and a non-significant effect on time to full RTW (HR = 1.29, 95% CI = 0.91–1.81) (very low certainty).

#### Secondary outcomes

The 20 included studies investigated several of our pre-specified secondary outcomes (see Table 1). We extracted outcome data on self-efficacy, change in symptoms (depression, pain, health-related quality of life, fear avoidance beliefs, kinesiophobia), physical-, cognitive- and social function, and cost-effectiveness. None of the studies assessed work motivation. Appendix 4 shows the results of the secondary outcomes. All outcomes had very low certainty, mostly due to imprecision and risk of bias. Some single studies demonstrate the effect on different health-related outcomes, such as pain, health complaints, depression, and physical function [34, 37, 41, 45]. The certainty of evidence for the secondary outcomes is presented in Appendix 4.

## Discussion

This review included 20 RCTs from 2000 to 2020 that examined different types of return to work interventions for people on sick leave in Denmark, the Netherlands, Norway, and Sweden. Very low- to moderate- certainty evidence suggested mostly no or marginal benefits of the return to work interventions. This is in line with a dozen related systematic reviews published since 2012 [55–63]. These reviews had different inclusion criteria and included between 2 and 50 studies (average 23).

Much of the reason for the very low- to moderate- certainty evidence was the inconsistent and imprecise effect estimates among the included studies. While half of the studies assessed multidisciplinary rehabilitation, the studies were heterogeneous in terms of interventions and comparisons. Thus, most comparisons were made up of only one RCT investigating a specific intervention. Also, the studies in the meta-analyses varied somewhat in content and design, although the basic principles of the multidisciplinary rehabilitation were similar. The included studies sampled participants on 1-24 months of sick leave, but with quite a dissimilar length of sick leave among the studies. According to Norwegian sick leave data, the length of sick leave might influence the ability to achieve differences in RTW among the intervention and control groups [64]. The rate for RTW between weeks 2 and 8 is generally high, while the RTW rate gradually decreases between weeks 12 and 52 [64]. Also, it varies among countries how much and how long the economic support for sick leave absentees is provided. For example, there is 12 months of support in Norway, Sweden, and Denmark and 24 months in the Netherlands, which might influence the RTW rate after 12 months of follow-up [3]. Of note, many of our included studies had relatively small sample sizes, and we acknowledge that the absence of significant results might be due to a lack of power. In general, the studies had a low risk of bias across most domains, but all except one study had no blinding of participants and personnel. Acknowledging the importance of blinding to avoid performance bias, we considered this a limitation despite RTW being an objectively assessed outcome.

We detected some promising results, whereby a few comparisons consisting of a few or only one study, showed some effect for the outcome RTW [31, 34, 41]. Especially one study with a comprehensive multidisciplinary rehabilitation program compared to ACT would be useful for further investigations in different populations and settings [31]. Also, the four studies assessing W-CBT showed some effect compared to the control groups [35, 43], although the results varied in terms of the RTW rate [42, 51, 52]. Other systematic reviews examining various psychological treatments have found some effects on RTW and demonstrate a potential to find an effective treatment for people on sick leave [56, 61, 65]. Several of our included interventions were based on previously investigated programs, and they either confirmed earlier, promising results [50], or failed to reproduce earlier results [37, 44].

The included studies also assessed several of our secondary outcomes. They detected minimal effects on symptom reduction, physical/social/cognitive function, and cost-effectiveness. A possible explanation might be that the interventions often were compared to other active interventions with a health-related approach. Similar systematic reviews assessing symptom outcomes, such as depression and anxiety, have found no significant effect in people with CMDs [56, 57, 61].

The issue of generalizability and whether a work-related intervention should be considered implemented in other countries or settings is a complicated one in this situation. The 20 trials were from four northern European countries only and fourteen of these trials were from Scandinavia, which, like the Netherlands, are known for their economic strength and societal welfare. They rank among the top 17 countries listed by GDP per capita and are known for their free market economies heavily taxed to support broad-reaching welfare states. Still, while most of the included studies were conducted in Scandinavia, the problem of partial or full sick leave is encountered in many other countries. We acknowledge that in work-related interventions, the results may be affected by a multitude of factors, such as the interests of the company, workers’ ability and motivation to minimize sickness absence, differences in sick leave arrangements, such as compensation and economy, time allowed on sick leave, and job security.

The effectiveness of work-related interventions has relevance for the societal perspective, such as sickness absentees, social and health care providers, and policy makers. An important aspect is the cost-effectiveness of an intervention to help reduce the societal cost of sickness absence. Our results might give some insight to the challenges and possibilities for policy makers in vocational rehabilitation. Seven of the ten comparisons in this review were supported by only one RCT with a limited number of participants. Replication studies of the promising interventions [31, 34, 35, 43, 50], should be carried out to assess the effectiveness and generate greater certainty of the evidence. It is critical that future studies have a low risk of bias—cluster-randomized controlled trials and pre-randomization of participants could be considered to address the issue of blinding—are adequately powered, and have detailed reporting of both methods and results.

Our systematic review comes with strengths and limitations. The greatest strength is the systematic approach, including extensive literature searches, and that we only included RCTs, of which most had a low risk of bias. With adjusted inclusion criteria, such as additional study designs and shorter sick leave duration, more studies would have been included. However, as indicated by the results of previous related reviews [55, 60, 61], the certainty of the evidence would likely remain unchanged. Although the systematic review was performed by researchers specializing in systematic review research and the searches were conducted by a search specialist, it is possible that relevant studies have been missed and relevant studies may have been published after our last search. Our ability to conduct meta-analyses was limited—given study heterogeneity, inconsistent measurement, and reporting—as was our ability to conduct sensitivity analyses. As a result, it was neither possible to improve the precision to any great extent, nor statistically assess potential differences across groups, such as diagnoses, intervention dosage, length of sick leave, or geographical settings.

## Conclusion

This systematic review confirms the results of previous reviews of mostly no or marginal benefits of return to work interventions for people on long-term sick leave. Thus, despite including evidence from 20 RCTs from Denmark, the Netherlands, Norway, and Sweden, it is hitherto inconclusive what are the most effective return to work interventions for people on long-term sick leave. However, a handful of potential interventions exist, that may contribute to a foundation for future research. Our findings support the need for adequately powered and methodologically strong studies. We encourage researchers to thoroughly describe the interventions to enable comparisons and implementation into practice.

## Supplementary Information

**Additional file 1: Appendix 1**. Protocol. **Appendix 2**. Search strategy. **Appendix 3**. Excluded studies and reason. **Appendix 4**. Certainty of evidence for secondary outcomes.

## Data Availability

All relevant data are within the manuscript and its supporting information files.

## Abbreviations

CI: Confidence interval
CMD: Common mental disorders
GRADE: Grading of Recommendations Assessment, Development, and Evaluation
MD: Mean difference
MR: Multidisciplinary rehabilitation
NIPH: Norwegian Institute of Public Health
RCT: Randomized controlled trial
RR: Risk ratio
RTW: Return to work
SMD: Standardized mean difference
TIDieR: The template for intervention description and replication
UC: Usual care
W-CBT: Work-focused cognitive behavioral therapy
