# Supplementary material for: Effectiveness of work-related interventions for return to work in people on sick leave: a systematic review and meta-analysis of randomized controlled trials
Source: Syst Rev. 2022 Sep 5;11:192. doi: 10.1186/s13643-022-02055-7 (PMC9446672; doi:10.1186/s13643-022-02055-7)
Supplement: Supplementary file 1 — Additional file 1: Appendix 1. Protocol. Appendix 2. Search strategy. Appendix 3. Excluded studies and reason. Appendix 4. Certainty of evidence for secondary outcomes. [file 13643_2022_2055_MOESM1_ESM.docx]

Appendix/supplement

1. Protocol
2. Search strategy
3. Excluded studies and reason
4. Certainty of evidence for secondary outcomes

**Appendix 1. Protocol**

# Protocol for Work-related interventions for people on long-term sick leave

| **Protocol established**: | January-March 2020 (in Norwegian (1)) |
| --- | --- |
| **Translated:** | December 2020 (English) |

The Norwegian Institute of Public Health (NIPH) was commissioned by the Norwegian Labour and Welfare Administration (NAV) to conduct a systematic review on the effect of work-related interventions for people on long-term sick leave and people at risk for long-term sick leave.

**Description**

In the present *Letter of Intent regarding a more inclusive working life* (“IA agreement”), the two main objectives are to reduce sick leave and to reduce withdrawal from work life. The latter relates particularly to people on long-term sick leave who might not return to work. To this group the Labour and Welfare service (NAV) and the health services offer various occupational rehabilitation interventions, however, the effects of these interventions remains uncertain. NAV commissioned the Norwegian Institute of Public Health to carry out a systematic review about the studies of effect of work-related interventions among people on long-term sick leave or at risk for long-term sick leave. We will primarily look at effects on return to work on short and long term, secondarily at effects on self-efficacy, disease symptoms, function and cost-effectiveness.

| **Project category** | |
| --- | --- |
| **Product:** | Systematic review |
| **Thematic area:** | Work and welfare |
| **Commissioner**: | Norwegian Labour and Welfare Administration (NAV) |
| **Project management and participants** | |
| **Project leader:** | Alexander Tingulstad |
| **Responsible for the project**: | Rigmor C. Berg |
| **Internal project participants:** | Line Holtet Evensen, Maria Bjerk, Jose F. Meneses-Echavez, Hilde H. Holte, Gyri Hval |
| **Internal peer review:** | Hege Kornør, Jan Peter William Himmels |
| **External peer review:** | Hege Randi Eriksen (Western Norway University of Applied Sciences), Chris Jensen (Nasjonal kompetansetjeneste for arbeidsrettet rehabilitering) |
| **Expert group:** | Gunn Hege Marchand (The Norwegian University of Science and Technology, NTNU),  Randi Wågø Aas (Oslo Metropolitan University, University of Stavanger) |
| **Plan for replacement if project participants drop out:** | The leaders will find replacements |

### Aim

This systematic review aims to identify, assess and summarize available research about the effects of work-related interventions for people on long-term sick leave and those at risk of long-term sick leave. Findings from this review will contribute to evidence-informed policy making for the welfare services.

### Background

Long-term sick leave is a serious concern in developed countries, including Norway (2). The last years, national measures have been implemented to reach a more inclusive working life. The aim of these measures is to prevent sick leave and to reduce the falling out from working life. A report from 2018 found that there is still a lack of knowledge regarding the effects of interventions that is supposed to reduce sick leave (3). The authors reported that the work-related rehabilitation interventions assessed in Norway have shown varied results (3).

*Sick leave in Norway*

The total season-adjusted sick leave was 5.9 percent in the third quarter in 2019 (4). The last years the proportion of sick leave has been relatively stable around 6 percent (4). There has been additional focus the previous years to prevent sick leave in areas such as hospitals, nursing homes, kinder gardens, oil industry, public transports, and entrepreneur industries (4).

Divided on gender, the total sick leave for men were 4.5 percent and for women 7.5 percent, which is similar to other countries (4). There is a tendency to shorter periods of sick leave for men, while the women are prone to long term sick leave. Previous research has debated the complex relationship between traditional types of occupation for women, responsibility for the family and at home might influence sick leave (5).

Musculoskeletal disorders were the most common reason for sick leave with 33 percent of all sick notes in the third quarter of 2019 (4). Common mental disorders came second with 17 percent. However, research has shown that the lines between these diagnoses are thin, and that common mental disorders are underreported, and often disguised as a musculoskeletal diagnosis (4). Respiratory diseases are placed as the third most common cause of sick-leave with 13 percent.

Most sick leave episodes were short, 60 percent lasted 16 days or shorter. A total of 80 percent was shorter than 8 weeks, while 20 percent of the periods was for more than 8 weeks, and 8 percent lasted 6 months or more (4). After 12 months, the rights to sick pay reaches it limit, and those still on sick leave must apply for other disability benefits. Statistics from NAV show that the probability of returning to work is highest during the first weeks, thereafter the probability decreases significantly, before it increases again from 9 months to the end of the sick leave period of 12 months (6).

Context in Norway

All legal residents in Norway are included in the national insurance scheme and have the right to 100% paid sick leave for up to 12 months. The first 16 calendar days are paid by the employer, before the government (NAV) pays the remaining period. The sick pay corresponds to the full wage, up to a certain limit. After one year, the worker must either return to work or apply for other welfare benefits (e.g., disability pension).
In comparison to other similar countries, Norway has a higher proportion of people on sick leave. This might be influenced by several factors, such as high work participation and good sick leave arrangements (7, 8). These factors vary in other countries, with regards to length of benefits, percentage of salary covered by the benefits, the possibility of losing job while on sick leave and low work participation (7-10). Hence, comparison of somewhat similar countries might be challenging in the area of work and welfare.

Work-related rehabilitation

These interventions should accompany the employer to facilitate return to work after sick leave or help people at risk of sick leave stay at work. In Norway, work-related interventions can be a collaboration between NAV and the health services as well as the employer, regardless of being public or private.

The aim of work-related rehabilitation is to strengthen the work-ability, emphasize work self-efficacy, and to overcome obstacles for work participation (10). The interventions vary, and may be intensive with shorter or longer stays at rehabilitation centers, or relevant treatment and education. If appropriate, the interventions can be intensive with either shorter or longer stays at rehabilitation centers, or relevant treatment and education. Those might include group or individual follow-up, physiotherapy, physical exercise, stress-coping, vocational guidance, and cooperation with employers.

There are uncertainty and partly disagreements among experts regarding the effect on return to work of such interventions (3). A systematic review assessing the effects of work-related rehabilitation for people on sick leave might contribute to a consensus among policy-makers and experts in the field.

### Methods

We plan to conduct a systematic review of primary research according to the NIPHs established methodological handbook (11). In cooperation with the NAV, we have decided not to conduct an umbrella review, as to the lack of details regarding the interventions and the populations will limit the use of the results. Besides including primary studies, we will include systematic reviews of high quality. Summaries from those reviews will be listed in the final report. Potential adjustments to the protocol during the project will be discussed with NAV.

###### a) Search strategy

We will search in the following databases:

- Campbell Collaboration (Subject area: Social Welfare)
- Cochrane Database of Systematic Reviews
- Epistemonikos (Broad Synthesis & Systematic Reviews)
- Cochrane Central Register of Controlled Trials
- Embase
- MEDLINE
- PsycINFO
- Scopus
- Sociological Abstracts (incl. Social Services Abstracts)
- SveMed+

A research librarian will develop a search strategy in cooperation with the project leader; the search and strategy will be peer reviewed by another information specialist. The research librarian will run the searches. The final strategy will be published as appendix in the final report.

We will also search grey literature in search engines like Google Scholar and web pages of relevant Scandinavian institutions. Additionally, reference lists will be inspected by one reviewer.

###### b) Inclusion and exclusion criteria

The following questions are to be answered: What are the effects of work-related rehabilitation for people on long-term sick leave and those who are at risk of becoming long-term sick leaved? The following inclusion criteria are developed in cooperation with NAV.

| *Inclusion criteria* | |  |
| --- | --- | --- |
| *Popula­tion* | - 1) Employees with partial or full sick leave for a period of time of 1-24 months, OR Employees that are at risk of long-term sick leave (less than 30% of the population OR   People that are not employed are included if less than 30% of the population AND   - 2) Are on long term sick leave (1-24 months) due to any diagnosis | |
| *Intervention* | - Health intervention with the aim of return to work, meaning it includes one or more components aimed at work, OR - Work-related interventions with an active health component - If a work-related intervention combines a health component and a non-health component, the study is included if the work-related intervention with a health component accounts for >70 % of the intervention. | |
| *Comparison* | - Usual care, other or no intervention | |
| *Outcome* | - 1) Primary outcome: full or partial return to work, time until return to work, time at work before new period of sick leave - 2) Secondary outcomes: self-efficacy, work motivation, symptom reduction, physical/social/cognitive function, cost-effectiveness | |
| *Study design* | - Primary studies with the following study design: randomized controlled trials (RCT), non-randomized controlled trials and controlled studies. If we identify several high quality RCTs, we will possibly only include RCTs. - In addition, we will include systematic reviews of high methodological quality. Methodological quality will be assessed with checklists. | |
| *Context* | - If we identify a high number of studies, we may limit inclusion to studies conducted in countries deemed as generalizable to Norway. | |
| *Language* | - We will include studies in languages that project participants or close colleagues master, such as English, German, Spanish, Portuguese, Italian, French, Finnish, Danish, Swedish and Norwegian. | |

| *Year* | - Primary studies published in 2000 and later - Systematic reviews published in 2010 or later |
| --- | --- |

| *Exclusion criteria* | |
| --- | --- |
| *Population* | - People with psychotic disorders |
| *Intervention* | - Individual Placement and Support or Supported Employment - Interventions at the workplace, without a health component - Health intervention, without any work-related components - Interventions with only preventive components, >30% of the participants are not on sick leave, only at risk |
| *Study ­design* | - Uncontrolled studies, non-systematic reviews/narrative reviews, qualitative studies, non-empirical studies, cohort studies and observational studies |

###### c) Study selection

The identified references will be imported to EndNote. We will use EPPI-Reviewer to select relevant studies. Two researchers will independently select relevant references based on title and abstract. Included studies will be assessed in full text by two independently researchers and included if relevant. All disagreements will be solved by discussion or, if needed, by involving a third reviewer.

###### d) Assessment of risk of bias/study quality

Two independent reviewers will appraise the systematic reviews by using NIPHs checklist based on the EPOC Checklist for Refereeing Protocols for Reviews or Amstar-2 for the assessment of methodological quality of systematic reviews (11, 12). Disagreements will be solved by discussion or by involvement of a third researcher.

If we include many high quality RCTs, we will consider to only include RCTs. To assess the methodical quality, we will use check lists. For RCTs, we will use Cochranes Risk of Bias tool (13). For non-RCTs, we will use Cochranes *Effective Practice and Organisation of Care* (EPOC) checklist (14). Two independent researchers will appraise all studies, and disagreements will be solved by discussion or by involvement of a third researcher.

###### e) Data extraction and analysis

*Data extraction*

One researcher will extract data from the included primary studies and another researcher will check the extracted data for correctness. Disagreements will be solved by discussion, or involvement by a third researcher. We will extract the following data: author, year, study aim, title, purpose, number of participants, details regarding the population, context, intervention, comparisons, results, follow-up period, and attrition. For the outcome cost-effectiveness, we will not do our own analyses, but present the results narratively supported by tables.

Completeness of reporting of the work-related interventions will be evaluated and reported in accordance to the Template for Intervention Description and Replication (TIDieR) checklist (15). Regarding the systematic reviews of high-quality, we will only report the abstract, PICO, and main results.

Data synthesis

In this systematic review we will either synthesize the results statistically or narratively.

In a statistical analysis, we will analyze dichotomous outcome measures by calculating the relative risk (RR) and the 95% confidence interval (CI). We will analyze continuous outcomes using the mean difference (MD) with 95% CI, or standardized mean difference (SMD), if the outcome measures have different units or scales of measurements. We will perform meta-analyses if primary studies have the same outcomes and are sufficiently similar in terms of population, intervention, comparison, and effect measurement, using random effects models (16). Whether the studies and PICO are similar enough will be assessed when the data are available. We might expect that the interventions have different effects in different contexts and populations, and we therefore aim to find a mean effect. We will use RevMan 2014 software to generate forest plots to display measurement results.

We will initially inspect graphs visually to investigate the possibility of statistical heterogeneity and then we will investigate heterogeneity between studies by considering the I² statistic alongside the Chi² P value (16). We will consider values of P < 0.1 to be indicative of significant heterogeneity. We will interpret an I² estimate greater than or equal to 50% and accompanied by a statistically significant Chi² statistic as evidence of substantial heterogeneity (Cochrane Handbook for Systematic Reviews of Interventions). If substantial heterogeneity is found in the primary outcome, we will explore reasons for heterogeneity. Thus, we will consider conducting sub group analyses on different populations and interventions in meta-analyses. Based on both sick-leave statistics and previous research, the potential aspects of interest are:

- Population (work category (4, 17), socio-economic status (18-21), gender (4, 5, 22), age (4, 17))
- Diagnoses (4)
- Time on sick leave at baseline (4, 6)
- Different types of work-related interventions (3)

In case a statistical synthesis is not appropriate, we will summarize the results narratively by presenting both text and tables. If we use a narrative synthesis, we will use suitable guidance from the literature (SWiM (23)) to perform a thorough and meaningful analysis.

###### f) Generalizability

Findings from this review will be used by the commissioner (NAV) and other stakeholders to inform policy making in Norway. We will assess whether review findings are generalizable to Norwegian contexts.

To determine this, we will assess the following:

1. Length and coverage of sickness benefits payment
2. The possibility of losing jobs while on sick leave
3. Form of the social system (health system, work and welfare system, workplaces)

If suitable, we will include these factors the analyzes, statistical or narrative.

###### g) Assessment of the certainty of evidence

We will use the GRADE approach (Grading of Recommendations Assessment, Development, and Evaluation) to assess the certainty of the evidence (24). We will assess the certainty for the documentation of each of the identified outcomes (25).

We will integrate analysis of quality of evidence and the magnitude of effect of the interventions in the “Summary of findings” tables. The GRADE approach considers the risk of bias and the body of literature to rate certainty on the evidence into one of four levels:

*High certainty*: We are very confident that the true effect lies close to that of the

meta-analysis result.

*Moderate certainty*: We are moderately confident in the meta-analysis result: The

true effect is likely to be close to the meta-analysis result, but there is a possibility

that it is substantially different.

*Low certainty*: Our confidence in the meta-analysis result is limited: The true effect may be substantially different from the meta-analysis result.

*Very low certainty*: We have very little confidence in the meta-analysis result: The

true effect is likely to be substantially different from the meta-analysis result.

The GRADE asessment will be performed by two researchers, where each of them controls the others grading. If disagreements occur, a third researcher will be involved in the discussion.

###### h) Peer review

The protocol and final report will be assessed by the commissioner (NAV) for questions and comments. We will ensure close communication with NAV throughout the conduct of the project. The protocol and report will also be reviewed by both internal and external peer reviewers with written feedback, and at the end the products are approved by the department leaders at NIPH.

###### i) Reference group

The project has a reference group with experts in the field, which will be contacted if issues arise during the project. They will also review the protocol and report when needed.

### Activities and schedule

Time schedule for the different assignments:

- Protocol: March 2020
- Literature search: April 2020
- Selection of studies: April-May 2020
- Data extraction and analyses: May-November 2020
- First draft to NAV: December 2020
- Complete report and peer review: January 2021
- Approved report and publication: February 2021

### Publication

A report will be presented in Norwegian at the web pages of NIPH. Afterwards it might be relevant to write a scientific article to an international journal, in English or Norwegian, possibly with contribution from reviewers, reference group or NAV.

### Related publications (in Norwegian) from the NIPH

Dalsbø TK, Knapstad M. Arbeidsplasstiltak får trolig flere sykmeldte tilbake på jobb. Oslo: rapport Folkehelseinstituttet; 2015.

[https://www.fhi.no/publ/2015/arbeidsplasstiltak-far-trolig-flere-sykmeldte-tilbake-pa-jobb/](https://www.fhi.no/publ/2015/arbeidsplasstiltak-far-trolig-flere-sykemeldte-tilbake-pa-jobb/)

Meneses-Echavez JF, Baiju N, Berg RC. Effekt av gradert sykmelding vs. full sykmelding på sykefravær og arbeidstilknytning. Oslo: rapport Folkehelseinstituttet; 2018. <https://www.fhi.no/publ/2018/effekt-av-gradert-sykmelding-vs.-full-sykmelding-pa-sykefravar-og-arbeidsti/>

Myrhaug HT, Nguyen LH. Råd og anbefalinger for samtidige helse- og arbeidsrettede

tiltak: Systematisk litteratursøk med sortering. Oslo: Folkehelseinstituttet, 2019.

<https://www.fhi.no/publ/2019/rad-og-anbefalinger-for-samtidige-helse--og-arbeidsrettede-tiltak/>

### References

1. Protocol. Protocol in Norwegian: Norwegian Institute of Public Health

2. OECD. Sickness, Disability and Work: Breaking the Barriers2010.

3. Faggruppen for IA-avtalen. Målene om et mer inkluderende arbeidsliv – status og utviklingstrekk. Rapport 2018.

4. Sundell T. Utviklingen i sykefraværet 3. kvartal 2019. NAV; 2019: NAV; 2019 [Available from: <https://www.nav.no/no/nav-og-samfunn/statistikk/sykefravar-statistikk/sykefravar>.

5. Ose SO, Jiang L, Bungum B. Det kjønnsdelte arbeidsmarkedet og kvinners arbeidshelse.: SINTEF, NTNU; 2014.

6. Nossen J, Brage S. Forløpsanalyse av sykefravær: Når blir folk friskmeldt? Arbeid og velferd 2016; (3); 2016.

7. Proba. Internasjonal sammenligning av sykefravær. Proba samfunnsanalyse; 2014.

8. OECD. Absence from work due to illness [Available from: <https://stats.oecd.org/index.aspx?queryid=30123>.

9. Ose S, Kaspersen S, Kalseth J. Sykefraværsoppfølging i Danmark, Finland, Island, Nederland, Norge, Sverige og Tyskland. SINTEF; 2018.

10. NorgesOffentligeUtredninger. Arbeid og inntektssikring. Tiltak for økt sysselsetting 2019 [Available from: <https://www.regjeringen.no/no/dokumenter/nou-2019-7/id2637967/>.

11. Folkehelseinstituttet. Slik oppsummerer vi forskning: Folkehelseinstituttet; 2014 [Available from: <https://www.fhi.no/kk/oppsummert-forskning-for-helsetjenesten/hva-er-en-kunnskapsoppsummering/>.

12. Shea BJ, Reeves BC, Wells G, Thuku M, Hamel C, Moran J, et al. AMSTAR 2: a critical appraisal tool for systematic reviews that include randomised or non-randomised studies of healthcare interventions, or both. BMJ. 2017;358:j4008.

13. CochraneCollaboration. Cochrane Handbook for Systematic Reviews of Interventions 2020 [Available from: <https://training.cochrane.org/handbook/current>.

14. EPOC. Suggested risk of bias criteria for EPOC reviews 2017 [Available from: <http://epoc.cochrane.org/sites/epoc.cochrane.org/files/public/uploads/Resources-for-authors2017/suggested_risk_of_bias_criteria_for_epoc_reviews.pdf>: .

15. Hoffmann TC, Glasziou PP, Boutron I, Milne R, Perera R, Moher D, et al. Better reporting of interventions: template for intervention description and replication (TIDieR) checklist and guide. BMJ : British Medical Journal. 2014;348:g1687.

16. Borenstein M, Hedges LV, Higgins JPT, Rothstein HR. Introduction to meta-analysis. West Sussex, UK: Wiley; 2009.

17. Spasova S, Bouget D, Vanhercke B. Sick pay and sickness benefit schemes in the European Union. European Commission. 2016.

18. Folkehelseinstituttet. Sosiale helseforskjeller i Norge. I: Folkehelserapporten - Helsetilstanden i Norge [Available from: <https://www.fhi.no/nettpub/hin/grupper/sosiale-helseforskjeller/>.

19. Roche A, Kostadinov V, Fischer J, Nicholas R, O'Rourke K, Pidd K, et al. Addressing inequities in alcohol consumption and related harms. Health Promot Int. 2015;30 Suppl 2:ii20-35.

20. Vinberg E, Karlsson L, Møller B, Ursin G, Larsen I. Sosial ulikehet, innvandring og kreft. En rapport on kreftforekomst etter landbakgrunn, utdanning, inntekt og bosted. Kreftregisteret. 2018.

21. Tikka C, Verbeek J, Tamminga S, Leensen M, Boer CAd. Rehabilitation and return to work after cancer. 2017.

22. Nossen JP. Kjønnsforskjellen i sykefravær: Hvor mye er det mulig å forklare med registerdata? Arbeid og velferd 2019;(4). 2019 [Available from: <https://www.nav.no/no/nav-og-samfunn/kunnskap/analyser-fra-nav/arbeid-og-velferd/arbeid-og-velferd/kjonnsforskjellen-i-sykefravaer-hvor-mye-er-det-mulig-a-forklare-med-registerdata>.

23. Campbell M, McKenzie JE, Sowden A, Katikireddi SV, Brennan SE, Ellis S, et al. Synthesis without meta-analysis (SWiM) in systematic reviews: reporting guideline. BMJ. 2020;368:l6890.

24. GRADE. The Grading of Recommendations Assessment DaE. GRADE Working Group; [Available from: <https://www.gradeworkinggroup.org/>.

25. CochraneCollaboration. Cochrane Handbook for Systematic Reviews of Interventions 2019 [Available from: <https://training.cochrane.org/handbook/current/chapter-08>.

**Appendix 2. Search strategy**

**Database: Ovid MEDLINE(R) and Epub Ahead of Print, In-Process, In-Data-Review & Other Non-Indexed Citations, Daily and Versions(R) 1946 to March 19, 2021**
Search date: 2021-03-22

1 *Sick Leave/ [hvp] (3496)

2 sick leave.ti. [hvp] (1180)

3 *Return to Work/ [hvp] (1905)

4 return to work.ti. [hvp] (2335)

5 ((sick leave or sickness abscence) adj3 (duration or reduc* or decreas*)).ti,ab,kf. (679)

6 or/1-5 [hvp] (7028)

7 Sick Leave/ (6025)

8 ((sick or medical) adj (leave or listed or listing)).ti,ab,kf. (6042)

9 ((work or job) adj absence).ti,ab,kf. (423)

10 (((sickness or illness or sick or medical) adj5 (absenteeism or absence)) or (long term sick or sick employee*)).ti,ab,kw. (6171)

11 or/7-10 (13507)

12 Return to Work/ (2838)

13 Rehabilitation, Vocational/ (9481)

14 return to work.ab,kf. (9225)

15 rtw.ab,kf. (1203)

16 work focused rehabilitation.ti,ab,kf. (6)

17 back to work.ti,ab,kf. (814)

18 (workability or work ability).ti,ab,kf. (2550)

19 ((workplace* or work site* or worksite* or job site* or jobsite*) adj3 (intervention or integration)).ti,ab,kf. (1135)

20 re-employment.ti,ab,kf. (192)

21 vocational rehabilitation.ti,ab,kf. (2525)

22 or/12-21 (23668)

23 11 and 22 (2333)

24 6 or 23 (7856)

25 (pretest-posttest study or pretesting or pre-post tests or quasi experimental design or quasi experimental study or quasi experimental study design).kw. or non-randomized controlled trials as topic/ or controlled before-after studies/ or randomized controlled trial.pt. or controlled clinical trial.pt. or multicenter study.pt. or pragmatic clinical trial.pt. or (randomis* or randomiz* or randomly).ti,ab. or groups.ab. or (trial or multicenter or multi center or multicentre or multi centre).ti. or (intervention? or effect? or impact? or controlled or control group? or (before adj5 after) or (pre adj5 post) or ((pretest or pre test) and (posttest or post test)) or quasiexperiment* or quasi experiment* or pseudo experiment* or pseudoexperiment* or evaluat*).ti,ab. (11238132)

26 24 and 25 (4498)

27 limit 26 to yr="2000 -Current" (4143)

28 "Systematic Review"/ or (meta-anal* or metaanal* or meta-regression* or overview of reviews or pubmed or medline or psycinfo).ti,ab. or ((systematic* or literature) adj3 (overview or review* or search*)).ti,ab. or meta-analysis.pt. or systematic review.kw. (707305)

29 24 and 28 (498)

30 limit 29 to yr="2010 -Current" (377)

31 27 or 30 (4208)

**Database: Embase 1974 to 2021 March 19**
**Search date: 2021-03-22**

1 *medical leave/ (2052)

2 sick leave.ti. (1331)

3 *return to work/ (1915)

4 return to work.ti. (2812)

5 ((sick leave or sickness abscence) adj3 (duration or reduc* or decreas*)).ti,ab,kw. (873)

6 or/1-5 (6252)

7 medical leave/ (7144)

8 ((sick or medical) adj (leave or listed or listing)).ti,ab,kw. (7748)

9 ((work or job) adj absence).ti,ab,kw. (593)

10 (((sickness or illness or sick or medical) adj5 (absenteeism or absence)) or (long term sick or sick employee*)).ti,ab,kw. (7936)

11 or/7-10 (16620)

12 return to work/ (7114)

13 vocational rehabilitation/ (8401)

14 return to work.ab,kw. (12319)

15 rtw.ab. (1515)

16 work focused rehabilitation.ti,ab,kw. (9)

17 back to work.ti,ab,kw. (1181)

18 (workability or work ability).ti,ab,kw. (3161)

19 ((workplace* or work site* or worksite* or job site* or jobsite*) adj3 (intervention or integration)).ti,ab,kw. (1362)

20 re-employment.ti,ab,kw. (216)

21 vocational rehabilitation.ti,ab,kw. (3550)

22 or/12-21 (27813)

23 11 and 22 (2654)

24 6 or 23 (7533)

25 exp crossover procedure/ (66617)

26 exp double blind procedure/ (183190)

27 exp single blind procedure/ (42459)

28 exp clinical trial/ (1589593)

29 exp randomized controlled trial/ (654803)

30 (randomis* or randomiz* or randomly or trial or intervention? or effect? or impact? or multicenter or multi center or multicentre or multi centre or controlled or control group? or quasiexperiment* or quasi experiment* or double blind* or single blind* or assign* or allocat* or volunteer* or crossover or cross over).ti,ab. (10830098)

31 or/25-30 (11279023)

32 24 and 31 (3674)

33 limit 32 to yr="2000 -Current" (3414)

34 limit 24 to ("reviews (maximizes sensitivity)" and yr="2010 -Current") (3112)

35 33 or 34 (4606)

36 limit 35 to embase (2104)

**Database: APA PsycInfo 1806 to March Week 3 2021**
**Search date: 2021-03-22**

1 *Employee Leave Benefits/ (935)

2 sick leave.ti. (273)

3 *reemployment/ (1310)

4 return to work.ti. (773)

5 ((sick leave or sickness abscence) adj3 (duration or reduc* or decreas*)).ti,ab. (153)

6 or/1-5 (2441)

7 Employee Leave Benefits/ (1187)

8 ((sick or medical) adj (leave or listed or listing)).ti,ab. (1628)

9 ((work or job) adj absence).ti,ab. (152)

10 (((sickness or illness or sick or medical) adj5 (absenteeism or absence)) or (long term sick or sick employee*)).ti,ab. (2274)

11 or/7-10 (4104)

12 reemployment/ (1562)

13 Vocational Rehabilitation/ (6096)

14 return to work.ab. (2467)

15 rtw.ab. (510)

16 work focused rehabilitation.ti,ab. (2)

17 back to work.ti,ab. (260)

18 (workability or work ability).ti,ab. (800)

19 ((workplace* or work site* or worksite* or job site* or jobsite*) adj3 (intervention or integration)).ti,ab. (699)

20 re-employment.ti,ab. (186)

21 vocational rehabilitation.ti,ab. (3624)

22 or/12-21 (11339)

23 11 and 22 (715)

24 6 or 23 (2704)

25 control*.tw. (720397)

26 random:.tw. (211405)

27 exp treatment/ (1085514)

28 experimental design/ (11745)

29 between groups design/ (357)

30 quantitative methods/ (3473)

31 quasi experimental methods/ (303)

32 repeated measures/ (700)

33 ("0400" or "0451" or "1800" or "2000").md. (2604471)

34 (pre-post or "pre test$" or pretest$ or posttest$ or "post test$" or (pre adj5 post)).ti,ab. (61662)

35 ("quasi-experiment$" or quasiexperiment$ or "quasi random$" or quasirandom$ or "quasi control$" or quasicontrol$ or ((quasi$ or experimental) adj3 (method$ or study or trial or design$ or controlled))).ti,ab,hw. (66473)

36 (effect or impact or trial or intervention).ti. (244297)

37 repeated measure*.ti,ab. (17759)

38 ((before adj5 after) or control group*).ti,ab. (140592)

39 or/25-38 (3413033)

40 24 and 39 (2438)

41 limit 40 to yr="2000 -Current" (2180)

42 limit 24 to ("reviews (maximizes sensitivity)" and yr="2010 -Current") (969)

43 41 or 42 (2215)

**Database: Campbell Library, Social Welfare**
**Search date: 2021-03-25**

<https://campbellcollaboration.org/component/jak2filter/?Itemid=1352&issearch=1&isc=1&category_id=101&ordering=publishUp>

**Database: Cochrane Database of Systematic Reviews (reviews)**
**Search date: 2021-03-22**

#1 sick-leave:ti 197

#2 "return to work":ti 313

#3 ((sick-leave or sickness-abscence) NEAR/3 (duration or reduc* or decreas*)):ti,ab,kw 256

#4 #1 or #2 or #3 663

#5 MeSH descriptor: [Sick Leave] this term only 560

#6 ((sick or medical) NEXT (leave or listed or listing)):ti,ab,kw 1681

#7 ((work or job) NEXT absence):ti,ab,kw 88

#8 ((sickness or illness or sick or medical) NEAR/5 (absenteeism or absence)) or long-term-sick or sick-employee*:ti,ab,kw 1116

#9 #5 or #6 OR #7 or #8 2538

#10 MeSH descriptor: [Return to Work] this term only 239

#11 "return to work":ab,kw 2118

#12 rtw:ab,kw 207

#13 "work focused rehabilitation":ti,ab,kw 4

#14 "back to work":ti,ab,kw 76

#15 (workability or "work ability"):ti,ab,kw 487

#16 ((workplace* or work-site* or worksite* or job-site* or jobsite*) NEAR/3 (intervention or integration)):ti,ab,kw 691

#17 re-employment:ti,ab,kw 18

#18 "vocational rehabilitation":ti,ab,kw 471

#19 #10 or #11 or #12 or #13 or #14 or #15 or #16 or #17 or #18 3534

#20 #9 and #19 617

#21 #4 or #20 with Cochrane Library publication date Between Jan 2010 and Dec 2021, in Cochrane Reviews 24

**Database: Cochrane Central Register of Controlled Trials, Cochrane Database of Systematic Reviews (protocols)**
**Search date: 2021-03-22**

#1 ((sick-leave or sickness-abscence) NEAR/3 (duration or reduc* or decreas*)):ti,ab,kw 256

#2 MeSH descriptor: [Sick Leave] this term only 560

#3 ((sick or medical) NEXT (leave or listed or listing)) 1816

#4 ((work or job) NEXT absence) 136

#5 ((sickness or illness or sick or medical) NEAR/5 (absenteeism or absence)) or (long-term-sick or sick-employee*) 1117

#6 #2 or #3 OR #4 or #5 2667

#7 MeSH descriptor: [Return to Work] this term only 239

#8 "return to work" OR "returns to work" OR "returned to work" OR "returning to Work" 2727

#9 rtw 223

#10 "work focused rehabilitation" 4

#11 "back to work" 85

#12 (workability or "work ability") 520

#13 ((workplace* or work-site* or worksite* or job-site* or jobsite*) NEAR/3 (intervention or integration)) 753

#14 re-employment 24

#15 "vocational rehabilitation" 535

#16 #7 or #8 or #9 or #10 or #11 or #12 or #13 or #14 or #15 4229

#17 #6 and #16 765

#18 #1 or #17 with Cochrane Library publication date Between Jan 2010 and Dec 2020, in Cochrane Protocols 21

#19 #1 or #17 with Cochrane Library publication date Between Jan 2000 and Dec 2021, in Trials 738

**Database: Epistomonikos**
**Search date: 2021-03-23**

(advanced_title_en:((((sick OR medical) AND (leave OR listing OR listed)) OR ((work OR job) AND absence) OR ((sickness OR illness OR sick OR medical) AND (absenteeism OR absence)) OR "long term sick" OR "sick employee" OR "sick employees")) OR advanced_abstract_en:((((sick OR medical) AND (leave OR listing OR listed)) OR ((work OR job) AND absence) OR ((sickness OR illness OR sick OR medical) AND (absenteeism OR absence)) OR "long term sick" OR "sick employee" OR "sick employees"))) AND (advanced_title_en:("return to work" OR rtw OR "work focused rehabilitation" OR "back to work" OR workability OR "work ability" OR ((workplace* OR work-site* OR "work site" OR "work sites" OR worksite* OR job-site* OR "job site" OR "job sites" OR jobsite*) AND (intervention OR integration)) OR re-employment OR "vocational rehabilitation") OR advanced_abstract_en:("return to work" OR rtw OR "work focused rehabilitation" OR "back to work" OR workability OR "work ability" OR ((workplace* OR work-site* OR worksite* OR "work site" OR "work sites" OR job-site* OR "job site" OR "job sites" OR jobsite*) AND (intervention OR integration)) OR re-employment OR "vocational rehabilitation")) [Filters: classification=systematic-review, protocol=no, min_year=2010, max_year=2021]

4 broad syntheses, 2 structured summaries, 109 systematic reviews

**Database: Sociological Abstracts, Social services Abstracts (ProQuest)**
**Search date: 2021-03-23**

((ti(((sick OR medical) AND (leave OR listing OR listed)) OR ((work OR job) AND absence) OR ((sickness OR illness OR sick OR medical) AND (absenteeism OR absence)) OR "long term sick" OR "sick employee" OR "sick employees") OR ab(((sick OR medical) AND (leave OR listing OR listed)) OR ((work OR job) AND absence) OR ((sickness OR illness OR sick OR medical) AND (absenteeism OR absence)) OR "long term sick" OR "sick employee" OR "sick employees")) AND (ti("return to work" OR "return to work" OR “returns to work” OR “returned to work” OR “returning to work” OR rtw OR "work focused rehabilitation" OR "back to work" OR workability OR "work ability" OR ((workplace* OR work-site* OR worksite* OR job-site* OR jobsite*) AND (intervention OR integration)) OR re-employment OR "vocational rehabilitation") OR ab("return to work" OR “returns to work” OR “returned to work” OR “returning to work” OR rtw OR "work focused rehabilitation" OR "back to work" OR workability OR "work ability" OR ((workplace* OR work-site* OR worksite* OR job-site* OR jobsite*) AND (intervention OR integration)) OR re-employment OR "vocational rehabilitation"))) OR (ti("sick leave") OR ti("return to work"))Limits applied 2000-2020

**Database: Scopus**
**Search date: 2021-03-24**

( TITLE-ABS-KEY ( ( ( ( sick OR medical ) AND ( leave OR listing OR listed ) ) OR ( ( work OR job ) AND absence ) OR ( ( sickness OR illness OR sick OR medical ) AND ( absenteeism OR absence ) ) OR "long term sick" OR "sick employee" OR "sick employees" ) ) ) AND ( TITLE-ABS-KEY ( "return to work" OR "returns to work" OR "returned to work" OR "vocational rehabilitation" OR "returning to work" OR rtw OR "work focused rehabilitation" OR "back to work" OR workability OR "work ability" OR ( ( workplace* OR work-site* OR worksite* OR job-site* OR jobsite* ) AND ( intervention OR integration ) ) OR re-employment ) ) AND ( TITLE-ABS-KEY ( random* OR controlled OR control-group OR pre-test* OR post-test* OR pretest* OR posttest* OR experiment* OR quasiexperiment* OR quasirandom* OR quasicontrol* OR effect OR impact OR trial OR intervention OR ( before AND after ) ) ) AND ( LIMIT-TO ( PUBYEAR , 2021 ) OR LIMIT-TO ( PUBYEAR , 2020 ) OR LIMIT-TO ( PUBYEAR , 2019 ) OR LIMIT-TO ( PUBYEAR , 2018 ) OR LIMIT-TO ( PUBYEAR , 2017 ) OR LIMIT-TO ( PUBYEAR , 2016 ) OR LIMIT-TO ( PUBYEAR , 2015 ) OR LIMIT-TO ( PUBYEAR , 2014 ) OR LIMIT-TO ( PUBYEAR , 2013 ) OR LIMIT-TO ( PUBYEAR , 2012 ) OR LIMIT-TO ( PUBYEAR , 2011 ) OR LIMIT-TO ( PUBYEAR , 2010 ) OR LIMIT-TO ( PUBYEAR , 2009 ) OR LIMIT-TO ( PUBYEAR , 2008 ) OR LIMIT-TO ( PUBYEAR , 2007 ) OR LIMIT-TO ( PUBYEAR , 2006 ) OR LIMIT-TO ( PUBYEAR , 2005 ) OR LIMIT-TO ( PUBYEAR , 2004 ) OR LIMIT-TO ( PUBYEAR , 2003 ) OR LIMIT-TO ( PUBYEAR , 2002 ) OR LIMIT-TO ( PUBYEAR , 2001 ) OR LIMIT-TO ( PUBYEAR , 2000 ) )

( TITLE-ABS-KEY ( "return to work" OR "returns to work" OR "returned to work" OR "vocational rehabilitation" OR "returning to work" OR rtw OR "work focused rehabilitation" OR "back to work" OR workability OR "work ability" OR ( ( workplace* OR work-site* OR worksite* OR job-site* OR jobsite* ) AND ( intervention OR integration ) ) OR re-employment ) AND TITLE-ABS-KEY ( ( ( sick OR medical ) AND ( leave OR listing OR listed ) ) OR ( ( work OR job ) AND absence ) OR ( ( sickness OR illness OR sick OR medical ) AND ( absenteeism OR absence ) ) OR "long term sick" OR "sick employee" OR "sick employees" ) AND TITLE-ABS-KEY ( meta-anal* OR metaanal* OR meta-regression* OR overview-of-reviews OR pubmed OR medline OR psycinfo OR ( ( systematic* OR literature ) AND ( overview OR review* OR search* ) ) ) ) AND ( LIMIT-TO ( PUBYEAR , 2021 ) OR LIMIT-TO ( PUBYEAR , 2020 ) OR LIMIT-TO ( PUBYEAR , 2019 ) OR LIMIT-TO ( PUBYEAR , 2018 ) OR LIMIT-TO ( PUBYEAR , 2017 ) OR LIMIT-TO ( PUBYEAR , 2016 ) OR LIMIT-TO ( PUBYEAR , 2015 ) OR LIMIT-TO ( PUBYEAR , 2014 ) OR LIMIT-TO ( PUBYEAR , 2013 ) OR LIMIT-TO ( PUBYEAR , 2012 ) OR LIMIT-TO ( PUBYEAR , 2011 ) OR LIMIT-TO ( PUBYEAR , 2010 ) )

**Database: Google Scholar**
**Search date: 22.09.20**

"sick leave" OR "work absence" OR "job absence", 2000-2020 : 200

**Database: ORIA**
**Search date: 2020-06-11**

sykmeld* OR sykemeld* OR langtidssyk* OR "arbeidsrettede tiltak", 2000-2020, avhandlinger : 141 *(*[*Søkeresultat*](https://bibsys-almaprimo.hosted.exlibrisgroup.com/primo-explore/search?query=any,contains,sykmeld*%20OR%20sykemeld*%20OR%20langtidssyk*%20OR%20%22arbeidsrettede%20tiltak%22,AND&pfilter=pfilter,exact,dissertations,AND&pfilter=creationdate,exact,20-YEAR,AND&tab=default_tab&search_scope=blended_scope&sortby=rank&vid=SIRUS&lang=no_NO&mode=advanced&offset=0)*)*

sykmeld* OR sykemeld* OR langtidssyk* OR "arbeidsrettede tiltak", 2000-2020, artikler : 405 *(*[*Søkeresultat*](https://bibsys-almaprimo.hosted.exlibrisgroup.com/primo-explore/search?query=any,contains,sykmeld*%20OR%20sykemeld*%20OR%20langtidssyk*%20OR%20%22arbeidsrettede%20tiltak%22,AND&pfilter=pfilter,exact,articles,AND&pfilter=creationdate,exact,20-YEAR,AND&tab=default_tab&search_scope=blended_scope&sortby=rank&vid=SIRUS&lang=no_NO&mode=advanced&offset=0)*)*

sykmeld* OR sykemeld* OR langtidssyk* OR "arbeidsrettede tiltak", 2000-2020, masteroppgaver : 97 *(*[*Søkeresultat*](https://bibsys-almaprimo.hosted.exlibrisgroup.com/primo-explore/search?query=any,contains,sykmeld*%20OR%20sykemeld*%20OR%20langtidssyk*%20OR%20%22arbeidsrettede%20tiltak%22,AND&pfilter=pfilter,exact,masteroppgave,AND&pfilter=creationdate,exact,20-YEAR,AND&tab=default_tab&search_scope=blended_scope&sortby=rank&vid=SIRUS&lang=no_NO&mode=advanced&offset=0)*)*

**Database: Danbib**
**Search date: 2020-09-11**

sygeorlov, 2000-2020 : 58 *(*[*Søkeresultat*](https://bibliotek.dk/da/search/work?search_block_form=+sygeorlov&select_material_type=bibdk_frontpage&op=S%C3%B8g&year_op=%2522year_gt%2522&year_value=%25221999%2522&form_build_id=form-_kFbiG0eSf9vpbkLOFkVALXZxOSb9Zx-_eIiJ1hwOqk&form_id=search_block_form&sort=rank_frequency&page_id=bibdk_frontpage#content)*)*

langtidssygemelding, 2000-2020 : 4 *(*[*Søkeresultat*](https://bibliotek.dk/da/search/work?search_block_form=langtidssygemelding&select_material_type=bibdk_frontpage&op=S%C3%B8g&year_op=%2522year_eq%2522&year_value=&form_build_id=form-bg1csXCW6ylx9poHxx11GgH_Fr_iwsW2nwwm1NXzlDc&form_id=search_block_form&sort=rank_frequency&page_id=bibdk_frontpage#content)*)*

arbejdsorienterede foranstaltninger : 0

**Database: LIBRIS**
**Search date: 2020-09-11**

arbetsinriktade åtgärder, 2000-2020 : 0

långtidssjukskrivna, 2000-2020 : 138

sjukskriven, 2000-2020 : 153

**Appendix 3. Excluded studies and reason**

**Excluded studies after full-text assessment and reason for exclusion**

| **Study** | **Reason for exclusion** |
| --- | --- |
| Ahlstrom L, Hagberg M, Dellve L. Workplace rehabilitation and supportive conditions at work: a prospective study. J Occup Rehabil. 2013 Jun;23(2):248-60. | Study design |
| Andersen Å, Larsson K, Lytsy P, Berglund E, Kristiansson P, Anderzén I. Strengthened General Self-Efficacy with Multidisciplinary Vocational Rehabilitation in Women on Long-Term Sick Leave: a Randomised Controlled Trial. Journal of occupational rehabilitation 2018;28(4):691‐700. | Outcome measures |
| Anema JR, Steenstra IA, Bongers PM, De Vet HCW, Knol DL, Loisel P, et al. Multidisciplinary rehabilitation for subacute low back pain: Graded activity or workplace intervention or both? A randomized controlled trial. Spine 2007;32(3):291-8. | Population and intervention |
| Arnetz BB, Sjögren B, Rydéhn B, Meisel R. Early workplace intervention for employees with musculoskeletal-related absenteeism: a prospective controlled intervention study. Journal of occupational and environmental medicine 2003;45(5):499‐506. | Population and intervention |
| Arokoski JP, Juntunen M, Luikku J. Use of health-care services, work absenteeism, leisure-time physical activity, musculoskeletal symptoms and physical performance after vocationally oriented medical rehabilitation-description of the courses and a one-and-a-half-year follow-up study with farmers, loggers, police officers and hairdressers. International Journal of Rehabilitation Research 2002;25(2):119-31. | Study design |
| Aure OF, Nilsen JH, Vasseljen O. Manual therapy and exercise  therapy in patients with chronic low back pain: A randomized, controlled trial with 1-year follow-up. Spine 2003;28(6):525-31. | Intervention |
| Aybek S, Hubschmid M, Mossinger C, Berney A, Vingerhoets F. Early intervention for conversion disorder: Neurologists and psychiatrists working together. Acta Neuropsychiatrica 2013;25(1):52-6. | Intervention |
| Badii M, Keen D, Yu S, Yassi A. Evaluation of a comprehensive integrated workplace-based program to reduce occupational musculoskeletal injury and its associated morbidity in a large hospital. Journal of Occupational and Environmental Medicine 2006;48(11):1159-65. | Study design and population |
| Beaudreuil J, Kone H, Lasbleiz S, Vicaut T, Richette P, Cohen-Solal M, et al. Efficacy of a functional restoration program for chronic low back pain: Prospective 1-year study. Joint Bone Spine 2010;77(5):435-9. | Study design |
| Beiwinkel T, Eißing T, Telle NT, Siegmund-Schultze E, Rössler W. Effectiveness of a Web-Based Intervention in Reducing Depression and Sickness Absence: randomized Controlled Trial. J Med Internet Res 2017;19(6):e213. | Small proportion of participants on sick leave |
| Berglund E, Anderzen I, Andersen A, Carlsson L, Gustavsson C, Wallman T, et al. Multidisciplinary intervention and acceptance and commitment therapy for return-to-work and increased employability among patients with mental illness and/or chronic pain: A randomized controlled trial. International Journal of Environmental Research and Public Health 2018;15 (11) (no pagination)(2424). | Population and intervention |
| Bernacki EJ, Guidera JA, Schaefer JA, Tsai S. A facilitated early return to work program at a large urban medical center. J Occup Environ Med 2000;42(12):1172-7. | Study design |
| Bethge M, Herbold D, Trowitzsch L, Jacobi C. Work status and health-related quality of life following multimodal work hardening: a cluster randomised trial. J Back Musculoskeletal Rehabil 2011;24(3):161-72. | Population |
| Bethge M. [Work-Related Medical Rehabilitation]. Rehabilitation 2017;56(1):14- | Study design |
| Bitsch BL, Nielsen CV, Stapelfeldt CM, Lynggaard V. Effect of the patient education - Learning and Coping strategies - in cardiac rehabilitation on return to work at one year: a randomised controlled trial show (LC-REHAB). BMC Cardiovasc Disord. 2018 May 21;18(1):101. | Population and intervention |
| Björkelund C, Svenningsson I, Hange D, Udo C, Petersson EL, Ariai N, Nejati S, Wessman C, Wikberg C, André M, Wallin L, Westman J. Clinical effectiveness of care managers in collaborative care for patients with depression in Swedish primary health care: a pragmatic cluster randomized controlled trial. BMC Fam Pract. 2018 Feb 9;19(1):28. | Small proportion of participants on sick leave and intervention |
| Björneklett HG, Rosenblad A, Lindemalm C, Ojutkangas ML, Letocha H, Strang P, Bergkvist L. A randomized controlled trial of support group intervention after breast cancer treatment: results on sick leave, health care utilization and health economy. Acta Oncol. 2013 Jan;52(1):38-47. | Small proportion of participants on sick leave |
| Bogefeldt J, Grunnesjö MI, Svärdsudd K, Blomberg S. Sick leave reductions from a comprehensive manual therapy programme for low back pain: the Gotland Low Back Pain Study. Clin Rehabil. 2008 Jun;22(6):529-41. | Population and intervention |
| Bonde JP, Rasmussen MS, Hjøllund H, Svendsen SW, Kolstad HA, Jensen LD, Wieclaw J. Occupational disorders and return to work: a randomized controlled study. J Rehabil Med. 2005 Jul;37(4):230-5. | Intervention |
| Bouwsma EVA, Bosmans JE, van Dongen JM, Brölmann HAM, Anema JR, Huirne JAF. Cost-effectiveness of an internet-based perioperative care programme to enhance postoperative recovery in gynaecological patients: economic evaluation alongside a stepped-wedge cluster-randomised trial. BMJ Open. 2018 Jan 21;8(1):e017782. | Small proportion of participants on sick leave |
| Bouwsma EVA, Huirne JAF, van de Ven PM, Vonk Noordegraaf A, Schaafsma FG, Schraffordt Koops SE, van Kesteren PJM, Brölmann HAM, Anema JR. Effectiveness of an internet-based perioperative care programme to enhance postoperative recovery in gynaecological patients: cluster controlled trial with randomised stepped-wedge implementation. BMJ Open. 2018 Jan 30;8(1):e017781. | Small proportion of participants on sick leave |
| Braathen TN, Veiersted KB, Heggens J. Improved work ability and return to work following vocational multidisciplinary rehabilitation of subjects on long-term sick leave. Journal of Rehabilitation Medicine 2007;39(6):493-9. | Study design |
| Brämberg EB, Bergström G, Jensen I, Hagberg J, Kwak L. Effects of yoga, strength training and advice on back pain: a randomized controlled trial. BMC Musculoskelet Disord. 2017 Mar 29;18(1):132. | Population and intervention |
| Brouwers EP, de Bruijne MC, Terluin B, Tiemens BG, Verhaak PF. Cost-effectiveness of an activating intervention by social workers for patients with minor mental disorders on sick leave: a randomized controlled trial. Eur J Public Health. 2007 Apr;17(2):214-20. | Outcome measures |
| Brusco NK, Watts JJ, Shields N, Chan SP, Taylor NF. Does additional acute phase inpatient rehabilitation help people return to work? A subgroup analysis from a randomized controlled trial. Clin Rehabil. 2014 Aug;28(8):754-761. | Population and intervention |
| Bunketorp L, Lindh M, Carlsson J, Stener-Victorin E. The effectiveness of a supervised physical training model tailored to the individual needs of patients with whiplash-associated disorders--a randomized controlled trial. Clin Rehabil. 2006 Mar;20(3):201-17. | Intervention |
| Carlsson L, Englund L, Hallqvist J, Wallman T. Early multidisciplinary assessment was associated with longer periods of sick leave: a randomized controlled trial in a primary health care centre. Scand J Prim Health Care. 2013 Sep;31(3):141-6. | Intervention |
| Carrougher GJ, Brych SB, Pham TN, Mandell SP, Gibran NS. An Intervention Bundle to Facilitate Return to Work for Burn-Injured Workers: Report From a Burn Model System Investigation. J Burn Care Res. 2017 Jan/Feb;38(1):e70-e78. | Population and intervention |
| Dalgaard VL, Andersen LPS, Andersen JH, Willert MV, Carstensen O, Glasscock DJ. Work-focused cognitive behavioral intervention for psychological complaints in patients on sick leave due to work-related stress: Results from a randomized controlled trial. J Negat Results Biomed. 2017 Aug 22;16(1):13. | Outcome measures |
| de Buck PD, le Cessie S, van den Hout WB, Peeters AJ, Ronday HK, Westedt ML, Breedveld FC, Vliet Vlieland TP. Randomized comparison of a multidisciplinary job-retention vocational rehabilitation program with usual outpatient care in patients with chronic arthritis at risk for job loss. Arthritis Rheum. 2005 Oct 15;53(5):682-90. | Population |
| Durand MJ, Loisel P. Therapeutic Return to Work: Rehabilitation in the workplace. Work. 2001;17(1):57-63. PMID: 12441623. | Population |
| Ejeby K, Savitskij R, Ost LG, Ekbom A, Brandt L, Ramnerö J, Asberg M, Backlund LG. Symptom reduction due to psychosocial interventions is not accompanied by a reduction in sick leave: results from a randomized controlled trial in primary care. Scand J Prim Health Care. 2014 Jun;32(2):67-72. | Intervention |
| Eklund M, Wästberg BA, Erlandsson LK. Work outcomes and their predictors in the Redesigning Daily Occupations (ReDO) rehabilitation programme for women with stress-related disorders. Aust Occup Ther J. 2013 Apr;60(2):85-92. | Study design |
| Eklund M, Erlandsson LK. Women's perceptions of everyday occupations: outcomes of the Redesigning Daily Occupations (ReDO) programme. Scand J Occup Ther. 2014 Sep;21(5):359-67. | Study design |
| Ektor-Andersen J, Ingvarsson E, Kullendorff M, Orbaek P. High cost-benefit of early team-based biomedical and cognitive-behaviour intervention for long-term pain-related sickness absence. J Rehabil Med. 2008 Jan;40(1):1-8. | Population |
| Elvsashagen H, Tellnes G, Abdelnoor MH. Does early intervention by a specialist in physical medicine and rehabilitation reduce the duration of long term sick leave among persons with musculoskeletal diseases? Norsk Epidemiologi 2009;19(2):219-22. | Study design |
| Eshøj P, Tarp U, Nielsen CV. Effect of early vocational intervention in a rheumatological outpatient clinic--a randomized study. International journal of rehabilitation research Internationale Zeitschrift fur Rehabilitationsforschung Revue internationale de recherches de readaptation 2001;24(4):291‐7. | Not sufficient sick leave |
| Faux SG, Kohler F, Mozer R, Klein LA, Courtenay S, D'Amours SK, et al. The ROARI project - Road Accident Acute Rehabilitation Initiative: a randomised clinical trial of two targeted early interventions for road-related trauma. Clinical Rehabilitation 2015;29(7):639-52. | Not sufficient days on sick leave |
| Finnes A, Enebrink P, Sampaio F, Sorjonen K, Dahl J, Ghaderi A, et al. Cost-Effectiveness of Acceptance and Commitment Therapy and a Workplace Intervention for Employees on Sickness Absence due to Mental Disorders. Journal of Occupational and Environmental Medicine 2017;59(12):1211-20. | Intervention |
| Finnes A, Ghaderi A, Dahl J, Nager A, Enebrink P. Randomized controlled trial of acceptance and commitment therapy and a workplace intervention for sickness absence due to mental disorders. J Occup Health Psychol. 2019 Feb;24(1):198-212. | Intervention |
| Font MS, Rodilla JMR, Van Zon S, Ortega JA, Bultmann U, Pujadas CS. Effectiveness of a multi-faceted intervention to prevent musculoskeletal pain in nurses and aides: results of a cluster-randomized controlled trial. Occupational and environmental medicine 2019;76:A65‐. | Population and intervention |
| Forsbrand MH, Turkiewicz A, Petersson IF, Sennehed CP, Stigmar K. Long-term effects on function, health-related quality of life and work ability after structured physiotherapy including a workplace intervention. A secondary analysis of a randomised controlled trial (WorkUp) in primary care for patients with neck and/or back pain. Scandinavian Journal of Primary Health Care 2020;38(1):92-100. | Not sufficient sick leave |
| Framke E, Sørensen OH, Pedersen J, Rugulies R. Effect of a participatory organizational-level occupational health intervention on short-term sickness absence: A cluster randomized controlled trial. Scandinavian Journal of Work, Environment and Health 2016;42(3):192-200. | Not sufficient sick leave |
| Gjengedal RGH, Reme SE, Osnes K, Lagerfeld SE, Blonk RWB, Sandin K, Berge T, Hjemdal O. Work-focused therapy for common mental disorders: A naturalistic study comparing an intervention group with a waitlist control group. Work. 2020;66(3):657-667. | Study design |
| Godges JJ, Anger MA, Zimmerman G, Delitto A. Effects of education on return-to-work status for people with fear-avoidance beliefs and acute low back pain. Physical Therapy 2008;88(2):231-9. | Not sufficient sick leave |
| Grahn P, Palsdottir AM, Ottosson J, Jonsdottir IH. Longer nature-based rehabilitation may contribute to a faster return to work in patients with reactions to severe stress and/or depression. International Journal of Environmental Research and Public Health 2017;14 (11) | Intervention |
| Gross DP, Park J, Rayani F, Norris CM, Esmail S. Motivational Interviewing Improves Sustainable Return to Work in Injured Workers After Rehabilitation: A Cluster Randomized Controlled Trial. Archives of Physical Medicine and Rehabilitation 2017;98(12):2355-63. | Small proportion of participants on sick leave |
| Grossi G, Santell B. Quasi-experimental evaluation of a stress management programme for female county and municipal employees on long-term sick leave due to work-related psychological complaints. Journal of Rehabilitation Medicine 2009;41(8):632-8. | Intervention |
| Hagen EM, Eriksen HR, Ursin H. Does early intervention with a light mobilization program reduce long-term sick leave for low back pain? Spine 2000;25(15):1973-6. | Intervention |
| Hagen EM, Grasdal A, Eriksen HR. Does early intervention with a light mobilization program reduce long-term sick leave for low back pain: a 3-year follow-up study. Spine (Phila Pa 1976). 2003 Oct 15;28(20):2309-15; discussion 2316. | Intervention |
| Hagen EM. Does light mobilization treatment reduce long-term sick leave for low back pain? [Norwegian]. Norsk Epidemiologi 2006;16(2):137-44. | Intervention |
| Hagen EM, Ødelien KH, Lie SA, Eriksen HR. Adding a physical exercise programme to brief intervention for low back pain patients did not increase return to work. Scandinavian journal of public health 2010;38(7):731‐8. | Intervention |
| Haldorsen EM, Grasdal AL, Skouen JS, Risa AE, Kronholm K, Ursin H. Is there a right treatment for a particular patient group? Comparison of ordinary treatment, light multidisciplinary treatment, and extensive multidisciplinary treatment for long-term sick-listed employees with musculoskeletal pain. Pain. 2002 Jan;95(1-2):49-63. | Intervention |
| Hampel P, Tlach L. Cognitive-behavioral management training of depressive symptoms among inpatient orthopedic patients with chronic low back pain and depressive symptoms: A 2-year longitudinal study. Journal of Back and Musculoskeletal Rehabilitation 2015;28(1):49-60. | Intervention and outcome measures |
| Hampel P, Köpnick A, Roch S. Psychological and work-related outcomes after inpatient multidisciplinary rehabilitation of chronic low back pain: a prospective randomized controlled trial. BMC Psychol 2019;7(1):6. | Not sufficient sick leave and intervention |
| Hange D, Ariai N, Kivi M, Eriksson MC, Nejati S, Petersson EL. The impact of internet-based cognitive behavior therapy on work ability in patients with depression - a randomized controlled study. International journal of general medicine 2017;10:151-9. | Intervention |
| Hansen BB, Kirkeskov L, Begtrup LM, Boesen M, Bliddal H, Christensen R, et al. Early occupational intervention for people with low back pain in physically demanding jobs: A randomized clinical trial. PLoS Med 2019;16(8):e1002898. | Small proportion of participants on sick leave |
| Hara KW, Bjørngaard JH, Brage S, Borchgrevink PC, Halsteinli V, Stiles TC, et al. Randomized Controlled Trial of Adding Telephone Follow-Up to an Occupational Rehabilitation Program to Increase Work Participation. Journal of occupational rehabilitation 2018;28(2):265‐78. | Population |
| Hellstrom L, Bech P, Hjorthoj C, Nordentoft M, Lindschou J, Eplov LF. Effect on return to work or education of individual placement and support modified for people with mood and anxiety disorders: Results of a randomised clinical trial. Occupational and Environmental Medicine 2017;74(10):717-25. | Intervention and outcome measures |
| Hellström L, Madsen T, Nordentoft M, Bech P, Eplov LF. Trajectories of Return to Work Among People on Sick Leave with Mood or Anxiety Disorders: secondary Analysis from a Randomized Controlled Trial. Journal of occupational rehabilitation 2018;28(4):666‐77. | Intervention and outcome measures |
| Heymans MW, Vet HCW, Bongers PM, Koes BW, van Mechelen W. Back schools in occupational health care: design of a randomized controlled trial and cost-effectiveness study. Journal of manipulative and physiological therapeutics 2004;27(7):457‐65. | Not sufficient sick leave |
| Heymans MW, De Vet HCW, Bongers PM, Knol DL, Koes BW, Van Mechelen W. The effectiveness of high-intensity versus low-intensity back schools in an occupational setting: A pragmatic randomized controlled trial. Spine 2006;31(10):1075-82. | Not sufficient sick leave |
| Hlobil H, Staal JB, Twisk J, Köke A, Ariëns G, Smid T, et al. The effects of a graded activity intervention for low back pain in occupational health on sick leave, functional status and pain: 12-month results of a randomized controlled trial. Journal of occupational rehabilitation 2005;15(4):569‐80. | Intervention |
| Holzle P, Baumbach A, Mernyi L, Hamann J. Return to Work: A Psychoeducational Module - An Intervention Study. Psychiatrische Praxis 2018;45(6):299-306. | Intervention |
| Howe EI, Fure SCR, Løvstad M, Enehaug H, Sagstad K, Hellstrøm T, Brunborg C, Røe C, Nordenmark TH, Søberg HL, Twamley E, Lu J, Andelic N. Effectiveness of Combining Compensatory Cognitive Training and Vocational Intervention vs. Treatment as Usual on Return to Work Following Mild-to-Moderate Traumatic Brain Injury: Interim Analysis at 3 and 6 Month Follow-Up. Front Neurol. 2020 Nov 10;11:561400. | Intervention |
| Hubbard G, Gray NM, Ayansina D, Evans JM, Kyle RG. Case management vocational rehabilitation for women with breast cancer after surgery: a feasibility study incorporating a pilot randomised controlled trial. Trials [Electronic Resource] 2013;14:175. | Small proportion of participants on sick leave |
| Huichan-Munoz V, Justiniano-Cordero S, Solis-Hernandez JL, Rodriguez-Abrego G, Millan-Hernandez E, Rojano-Mejia D. [Return to work in patients with heart disease after cardiac rehabilitation]. Rev 2016;54(2):159-63. | Intervention |
| Høgelund J, Falgaard Eplov L. Employment effects of a multidisciplinary health assessment for mentally ill persons – A quasi-randomised controlled trial. Scandinavian Journal of Public Health 2018;46(3):389-99. | Intervention |
| Jensen C, Nielsen CV, Jensen OK, Petersen KD. Cost-effectiveness and cost-benefit analyses of a multidisciplinary intervention compared with a brief intervention to facilitate return to work in sick-listed patients with low back pain. Spine (Phila Pa 1976). 2013 Jun 1;38(13):1059-67. | Outcome measures |
| Jørgensen MB, Faber A, Hansen JV, Holtermann A, Søgaard K. Effects on musculoskeletal pain, work ability and sickness absence in a 1-year randomised controlled trial among cleaners. BMC public health 2011;11:840. | Small proportion of participants on sick leave |
| Karlson B, Jönsson P, Österberg K. Long-term stability of return to work after a workplace-oriented intervention for patients on sick leave for burnout. BMC public health 2014;14:821. | Intervention and study design |
| Van Der Klink JJL, Blonk RWB, Schene AH, Van Dijk FJH. Reducing long term sickness absence by an activating intervention in adjustment disorders: A cluster randomised controlled design. Occupational and Environmental Medicine 2003;60(6):429-37. | Intervention |
| Knapp S, Briest J, Bethge M. Work-related rehabilitation aftercare for patients with musculoskeletal disorders: results of a randomized-controlled multicenter trial. International journal of rehabilitation research Internationale zeitschrift fur rehabilitationsforschung Revue internationale de recherches de readaptation 2015;38(3):226‐32. | Outcome measures |
| Kool JP, Oesch PR, Bachmann S, Knuesel O, Dierkes JG, Russo M, et al. Increasing days at work using function-centered rehabilitation in nonacute nonspecific low back pain: a randomized controlled trial. Arch Phys Med Rehabil 2005;86(5):857-64. | Small proportion of participants on sick leave |
| Kool J, Bachmann S, Oesch P, Knuesel O, Ambergen T, de Bie R, et al. Function-centered rehabilitation increases work days in patients with nonacute nonspecific low back pain: 1-year results from a randomized controlled trial. Arch Phys Med Rehabil 2007;88(9):1089-94. | Small proportion of participants on sick leave |
| Kärrholm J, Ekholm K, Jakobsson B, Ekholm J, Bergroth A, Schüldt K. Effects on work resumption of a co-operation project in vocational rehabilitation. Systematic, multi-professional, client-centred and solution-oriented co-operation. Disability and Rehabilitation 2006;28(7):457-67. | Study design |
| Kärrholm J, Ekholm K, Ekholm J, Bergroth A, Ekholm KS. Systematic co-operation between employer, occupational health service and social insurance office: a 6-year follow-up of vocational rehabilitation for people on sick-leave, including economic benefits. Journal of rehabilitation medicine 2008;40(8):628‐36. | Intervention |
| Kääpä EH, Frantsi K, Sarna S, Malmivaara A. Multidisciplinary group rehabilitation Versus individual physiotherapy for chronic nonspecific low back pain: A randomized trial. Spine 2006;31(4):371-6. | Small proportion of participants on sick leave |
| Lagerveld SE, Blonk RWB, Brenninkmeijer V, de Meij LW, Schaufeli WB. Work-focused treatment of common mental disorders and return to work: A comparative outcome study. Journal of Occupational Health Psychology 2012;17(2):220-34. | Study design |
| Larsson UB, Kirkeby H, Nordström CH, Sjölund B. Rehabilitation of long-term sick-listed patients in Sweden through techniques of sports medicine. Journal of Back and Musculoskeletal Rehabilitation 2000;15(2-3):67-76. | Intervention and study design |
| Li EJQ, Li-Tsang CWP, Lam CS, Hui KYL, Chan CCH. The effect of a "training on work readiness" program for workers with musculoskeletal injuries: A randomized control trial (RCT) study. Journal of Occupational Rehabilitation 2006;16(4):529-41. | Outcome measures |
| Linton SJ, Boersma K, Jansson M, Svard L, Botvalde M. The effects of cognitive-behavioral and physical therapy preventive interventions on pain-related sick leave: A randomized controlled trial. Clinical Journal of Pain 2005;21(2):109-19. | Population and intervention |
| Lytsy P, Carlsson L, Anderzén I. Effectiveness of two vocational rehabilitation programmes in women with long-term sick leave due to pain syndrome or mental illness: 1-year follow-up of a randomized controlled trial. Journal of rehabilitation medicine 2017;49(2):170‐7. | Small proportion of participants were employed |
| Martin DJ, Chernoff RA, Buitron M, Scott Comulada W, Liang L, Wong F. Helping people with HIV/AIDS return to work: A randomized clinical trial. Rehabilitation Psychology 2012;57(4):280-9. | Small proportion of participants were employed |
| Nathell L. Effects on sick leave of an inpatient rehabilitation programme for asthmatics in a randomized trial. Scandinavian Journal of Public Health 2005;33(1):57-64. | Intervention |
| Netterstrøm B, Bech P. Effect of a multidisciplinary stress treatment programme on the return to work rate for persons with work-related stress. A non-randomized controlled study from a stress clinic. BMC public health 2010;10:658. | Study design |
| Nieuwenhuijsen K, Schoutens AMC, Frings-Dresen MHW, Sluiter JK. Evaluation of a randomized controlled trial on the effect on return to work with coaching combined with light therapy and pulsed electromagnetic field therapy for workers with work-related chronic stress. BMC Public Health 2017;17(1):761. | Intervention |
| Vonk Noordegraaf A, Anema JR, Van Mechelen W, Knol DL, Van Baal WM, Van Kesteren PJM, et al. A personalised eHealth programme reduces the duration until return to work after gynaecological surgery: Results of a multicentre randomised trial. BJOG: An International Journal of Obstetrics and Gynaecology 2014;121(9):1127-36. | Not sufficient days on sick leave |
| Noordik E, van der Klink JJ, Geskus RB, de Boer MR, van Dijk FJ, Nieuwenhuijsen K. Effectiveness of an exposure-based return-to-work program for workers on sick leave due to common mental disorders: a cluster-randomized controlled trial. Scand J Work Environ Health. 2013 Mar 1;39(2):144-54. | Intervention |
| Notenbomer A, Roelen C, Groothoff J, van Rhenen W, Bultmann U. Effect of an eHealth Intervention to Reduce Sickness Absence Frequency Among Employees With Frequent Sickness Absence: Randomized Controlled Trial. J Med Internet Res 2018;20(10):e10821. | Not sufficient days on sick leave |
| Nuechterlein KH, Subotnik KL, Ventura J, Turner LR, Gitlin MJ, Gretchen-Doorly D, et al. Enhancing return to work or school after a first episode of schizophrenia: the UCLA RCT of Individual Placement and Support and Workplace Fundamentals Module training. Psychol Med 2020;50(1):20-8. | Intervention |
| Nystuen P, Hagen KB. Feasibility and effectiveness of offering a solution-focused follow-up to employees with psychological problems or muscle skeletal pain: a randomised controlled trial. BMC Public Health 2003;3:19. | Outcome measures |
| Nystuen P, Hagen KB. Solution-focused intervention for sick listed employees with psychological problems or muscle skeletal pain: a randomised controlled trial. BMC public health 2006;6:69. | Intervention |
| Odeen M, Ihlebæk C, Indahl A, Wormgoor MEA, Lie SA, Eriksen HR. Effect of peer-based low back pain information and reassurance at the workplace on sick leave: A cluster randomized trial. Journal of Occupational Rehabilitation 2013;23(2):209-19. | Not sufficient sick leave |
| Oestergaard LG, Christensen FB, Bunger CE, Sogaard R, Holm R, Helmig P, et al. Does adding case management to standard rehabilitation affect functional ability, pain, or the rate of return to work after lumbar spinal fusion? A randomized controlled trial with two-year follow-up. Clinical Rehabilitation 2020;34(3):357-68. | Outcome measures |
| Park J, Esmail S, Rayani F, Norris CM, Gross DP. Motivational interviewing for workers with disabling musculoskeletal disorders: Results of a cluster randomized control trial. Journal of Occupational Rehabilitation 2018;28(2):252-64. | Population |
| Keus van de Poll M, Nybergh L, Lornudd C, Hagberg J, Bodin L, Kwak  L, Jensen I, Lohela-Karlsson M, Torgén M, Bergstrom G. Preventing sickness absence among employees with common mental disorders or stress-related symptoms at work: a cluster randomised controlled trial of a problem-solving-based intervention conducted by the Occupational Health Services. Occup Environ Med. 2020 Jul;77(7):454-461 | Small proportion of participants on sick leave |
| Rannard A, Gabbay M, Sen D, Riley R, Britt D. Feasibility trial of GP and case-managed support for workplace sickness absence. Primary Health Care Research and Development 2014;15(3):252-61. | Study design |
| Rasmussen CDN, Holtermann A, Jørgensen MB, Ørberg A, Mortensen OS, Søgaard K. A multi-faceted workplace intervention targeting low back pain was effective for physical work demands and maladaptive pain behaviours, but not for work ability and sickness absence: Stepped wedge cluster randomised trial. Scandinavian Journal of Public Health 2016;44(6):560-70. | Small proportion of participants on sick leave |
| Reme SE, Grasdal AL, Lovvik C, Lie SA, Overland S. Work-focused cognitive-behavioural therapy and individual job support to increase work participation in common mental disorders: a randomised controlled multicentre trial. Occup Environ Med 2015;72(10):745-52. | Intervention |
| Roche-Leboucher G, Petit-Lemanac'H A, Bontoux L, Dubus-Bausiére V, Parot-Shinkel E, Fanello S, et al. Multidisciplinary intensive functional restoration versus outpatient active physiotherapy in chronic low back pain: A randomized controlled trial. Spine 2011;36(26):2235-42. | Population |
| Salzwedel A, Wegscheider K, Schulz-Behrendt C, Dörr G, Reibis R, Völler H. No impact of an extensive social intervention program on return to work and quality of life after acute cardiac event: a cluster-randomized trial in patients with negative occupational prognosis. Int Arch Occup Environ Health. 2019 Nov;92(8):1109-1120. | Small proportion of participant were employed and unclear sick leave status |
| Scheenen ME, Visser-Keizer AC, de Koning ME, van der Horn HJ, van de Sande P, van Kessel M, et al. Cognitive Behavioral Intervention Compared to Telephone Counseling Early after Mild Traumatic Brain Injury: A Randomized Trial. Journal of Neurotrauma 2017;34(19):2713-20. | Population |
| Skouen JS, Grasdal AL, Haldorsen EMH, Ursin H. Relative cost-effectiveness of extensive and light multidisciplinary treatment programs versus treatment as usual for patients with chronic low back pain on long-term sick leave: Randomized controlled study. Spine 2002;27(9):901-9. | Intervention |
| Skouen JS, Grasdal A, Haldorsen EMH. Return to work after comparing outpatient multidisciplinary treatment programs versus treatment in general practice for patients with chronic widespread pain. European Journal of Pain 2006;10(2):145. | Outcome measures |
| Storro S, Moen J, Svebak S. Effects on sick-leave of a multidisciplinary rehabilitation programme for chronic low back, neck or shoulder pain: Comparison with usual treatment. Journal of Rehabilitation Medicine 2004;36(1):12-6. | Intervention |
| Streibelt M, Bethge M. Effects of intensified work-related multidisciplinary rehabilitation on occupational participation: a randomized-controlled trial in patients with chronic musculoskeletal disorders. International journal of rehabilitation research Internationale zeitschrift fur rehabilitationsforschung Revue internationale de recherches de readaptation 2014;37(1):61‐6. | Population |
| Streibelt M, Menzel-Begemann A. [Does the Success of Work-related Interventions in the Rehabilitation of Neurological Diseases Depend on the Return-to-Work Prognosis? A Re-analysis of 2 Randomised Controlled Trials]. Rehabilitation 2015;54(4):252-8. | Study design |
| Søgaard HJ, Bech P. The effect on length of sickness absence by recognition of undetected psychiatric disorder in long-term sickness absence. A randomized controlled trial. Scandinavian journal of public health 2009;37(8):864‐71. | Intervention |
| Taimela S, Malmivaara A, Justén S, Läärä E, Sintonen H, Tiekso J, et  al. The effectiveness of two occupational health intervention programmes in reducing sickness absence among employees at risk. Two randomised controlled trials. Occupational and environmental medicine 2008;65(4):236‐41. | Small proportion of participants on sick leave |
| Tamminga SJ, Verbeek JH, Bos MM, Fons G, Kitzen JJ, Plaisier PW, et al. Effectiveness of a hospital-based work support intervention for female cancer patients - a multi-centre randomised controlled trial. PLoS ONE [Electronic Resource] 2013;8(5):e63271. | Not sufficient sick leave |
| van de Leur, J.C., Buhrman, M., Åhs, F. *et al.* Standardized multimodal intervention for stress-induced exhaustion disorder: an open trial in a clinical setting. *BMC Psychiatry* **20,**526 (2020). | Study design |
| Verbeek JH, Van Der Weide WE, Van Dijk FJ. Early occupational health management of patients with back pain: A randomized controlled trial. Spine 2002;27(17):1844-51. | Not sufficient sick leave |
| Vermeulen SJ, Anema JR, Schellart AJM, Knol DL, Van Mechelen W, Van Der Beek AJ. A participatory return-to-work intervention for temporary agency workers and unemployed workers sick-listed due to musculoskeletal disorders: Results of a randomized controlled trial. Journal of Occupational Rehabilitation 2011;21(3):313-24. | Small proportion of participants were employed |
| Vikane E, Hellstrøm T, Røe C, Bautz-Holter E, Aßmus J, Skouen JS. Multidisciplinary outpatient treatment in patients with mild traumatic brain injury: a randomised controlled intervention study. Brain injury 2017;31(4):475‐84. | Population |
| Vlasveld MC, van der Feltz-Cornelis CM, Adre HJ, Anema JR, Hoedeman R, van Mechelen W. Collaborative care for sick-listed workers with major depressive disorder: a randomised controlled trial from the Netherlands Depression Initiative aimed at return to work and depressive symptoms. Occupational and environmental medicine 2012;70(4):223‐30. | Study design |
| Willert MV, Thulstrup AM, Bonde JP. Effects of a stress management intervention on absenteeism and return to work--results from a randomized wait-list controlled trial. Scand J Work Environ Health 2011;37(3):186-95. | Small proportion of participants on sick leave |
| Wormgoor MEA, Indahl A, Andersen E, Egeland J. Effectiveness of Briefer Coping-Focused Psychotherapy for Common Mental Complaints on Work-Participation and Mental Health: A Pragmatic Randomized Trial with 2-Year Follow-Up. Journal of Occupational Rehabilitation 2020;30(1):22-39. | Population |
| Wynne-Jones G, Artus M, Bishop A, Lawton SA, Lewis M, Jowett S, et al. Effectiveness and costs of a vocational advice service to improve work outcomes in patients with musculoskeletal pain in primary care: a cluster randomised trial (SWAP trial ISRCTN 52269669). Pain 2018;159(1):128‐38. | Population |
| Zaman ACGNM, Tytgat KMAJ, Klinkenbijl JHG, Boer FCD, Brink MA, Brinkhuis JC, Bruinvels DJ, Dol LCM, van Duijvendijk P, Hemmer PHJ, Lamme B, Loosveld OJL, Mok MM, Rejda T, Rutten H, Schoorlemmer A, Sonneveld DJ, Stassen LPS, Veenstra RP, van de Ven A, Velzing ER, Frings-Dresen MHW, de Boer AGEM. Effectiveness of a Tailored Work-Related Support Intervention for Patients Diagnosed with Gastrointestinal Cancer: A Multicenter Randomized Controlled Trial. J Occup Rehabil. 2021 Jun;31(2):323-338. | Not sufficient sick leave |
| Aakvik A, Holmas TH, Kjerstad E. A low-key social insurance reform--effects of multidisciplinary outpatient treatment for back pain patients in Norway. Journal of Health Economics 2003;22(5):747-62. | Study design |
| Aasdahl L, Pape K, Vasseljen O, Johnsen R, Gismervik S, Jensen C, et al. Effects of Inpatient Multicomponent Occupational Rehabilitation versus Less Comprehensive Outpatient Rehabilitation on Somatic and Mental Health: Secondary Outcomes of a Randomized Clinical Trial. Journal of Occupational Rehabilitation 2017;27(3):456-66. | Outcome measures |
| Aasdahl L, Gismervik SØ, Marchand GH, Vasseljen O, Johnsen R, Fimland MS. Changes in fear-avoidance beliefs and work  participation after occupational rehabilitation for musculoskeletal- and common mental disorders: Secondary outcomes of two randomized clinical trials. Journal of Rehabilitation Medicine 2019;51(3):175-82. | Study design and outcome measures |
| Aasdahl L, Pape K, Vasseljen O, Johnsen R, Fimland MS. Improved Expectations About Length of Sick Leave During Occupational Rehabilitation Is Associated with Increased Work Participation. Journal of Occupational Rehabilitation 2019;29(3):475-82. | Study design and outcome measures |

**Appendix 4. Certainty of evidence for secondary outcomes**

**Table: Certainty of evidence for secondary outcomes**

| Population: Adults on full or partly sick leave  Countries: Denmark, Norway, Sweden, The Netherlands  Intervention: Work-related treatment  Comparison: Treatment as usual or other active treatment | | | | | | |
| --- | --- | --- | --- | --- | --- | --- |
| Outcome, follow-up time | Relative effect  (95 % CI) | Anticipated absolute effects (95 % CI) | | | Number of participants  (studies) | Quality of evidence  (GRADE) |
|  |  | Assumed risk with control | Assumed risk with MR | Absolute difference (intervention minus control) |  |  |
| Multidisciplinary rehabilitation vs. UC |  |  |  |  |  |  |
| Change of symptoms: pain (2 mo) | - | - | - | One study reported reduction of pain in the intervention group compared to the control group | 38 participants (1 RCT) | ⨁◯◯◯ VERY LOW ^1,2^ |
| Change of symptoms: pain (3 mo) | - | - | - | One study reported less pain in the control group, and another study could not find any difference between the groups | 253 participants (2 RCTs) | ⨁◯◯◯ VERY LOW ^1,3^ |
| Change of symptoms: pain (6 mo) | - | - | - | No difference between the groups in level of pain | 172 participants  (2 RCTs) | ⨁◯◯◯ VERY LOW ^1,2^ |
| Change of symptoms: pain (12 mo) | - | - | - | No difference between the groups in level of pain | 291 participants  (3 RCTs) | ⨁◯◯◯ VERY LOW ^1,3^ |
| Change of symptoms: kinesiophobia (12 mo) | 1.71 Cohen’s *d* | - | - | Less kinesiophobia in the intervention group up to 12 months | 38 participants  (1 RCTs) | ⨁◯◯◯ VERY LOW ^1,2^ |
| Physical function (2 mo) | Cohen’s *d* = 0.92 | - | - | Better physical function in the intervention group | 38 participants  (1 RCTs) | ⨁◯◯◯ VERY LOW ^1,2^ |
| Physical function (3 mo) | - | - | - | No difference between the groups | 253 participants  (2 RCTs) | ⨁◯◯◯ VERY LOW ^1,3^ |
| Physical function (6 mo) | - | - | - | One study reported better physical function in the intervention group, while one study found no difference between the groups | 172 participants  (2 RCTs) | ⨁◯◯◯ VERY LOW ^1,2^ |
| Physical function (12 mo) | - | - | - | Two studies reported better physical function in the intervention group, while a third study found n difference between the groups | 291 participants  (3 RCTs) | ⨁◯◯◯ VERY LOW ^1,3^ |
| Cost-effectiveness | - | - | - | Two of the studies reported a cost-effective experimental intervention. One study had not a cost-effective experimental intervention | 291 participants  (3 RCTs) | ⨁◯◯◯ VERY LOW ^1,3^ |
| Multidisciplinary rehabilitation vs. active treatment |  |  |  |  |  |  |
| Change of symptoms: depression (12 mo) | MD = -0.72  ( -2.3, 0.9) | - | - | No difference between the groups | 166 participants (1 RCT) | ⨁◯◯◯ VERY LOW ^2,4^ |
| Change of symptoms: pain (12 mo) | - | - | - |  | 574 participants (3 RCTs) | ⨁⨁⨁◯ VERY LOW ^3,4^ |
| Change of symptoms: HRQoL (12 mo) | - | - | - | No difference between the groups | 404 participants (2 RCTs) | ⨁◯◯◯ VERY LOW ^3,4^ |
| Change of symptoms health complaints (12 mo) | - | - | - | No difference between the groups | 166 participants (1 RCT) | ⨁◯◯◯ VERY LOW ^2,4^ |
| Change of symptoms: fear avoidance behavior 12 mo) | Adjusted difference = −0.12 (−2.13, 1.90) ^†^ | - | - | No difference between the groups | 232 participants (1 RCT) | ⨁◯◯◯ VERY LOW ^3,5^ |
| Social function (12 mo) | Difference = 1.58 points (3.96-7.12) ^††^ | - | - | No difference between the groups | 232 participants (1 RCT) | ⨁◯◯◯ VERY LOW ^2,4^ |
| Physical function (12 mo) | . | - | - | No difference between the groups | 312 participants (2 RCTs) | ⨁◯◯◯ VERY LOW ^3,4^ |
| Cognitive function (12 mo) | MD = 4.64  (0.08-9.20) | - | - | Improved cognitive function in the experimental group | 240 participants (1 RCT) | ⨁◯◯◯ VERY LOW ^3,5^ |
| W-CBT vs. UC |  |  |  |  |  |  |
| Change of symptoms: depression (6 mo) | ANOVA F-value for the interaction effect = 0.12 | - | - | No difference between the groups | 72 participants (1 RCT) | ⨁◯◯◯ VERY LOW ^2,4^ |
| Change of symptoms: pain (6 mo) | ANOVA F-value for the interaction effect = 0.60 | - | - | No difference between the groups | 72 participants (1 RCT) | ⨁◯◯◯ VERY LOW ^2,4^ |
| Change of symptoms: physical function (6 mo) | ANOVA F-value for the interaction effect = 0.46 | - | - | No difference between the groups | 72 participants (1 RCT) | ⨁◯◯◯ VERY LOW ^2,4^ |
| W-CBT vs. active treatment |  |  |  |  |  |  |
| Change of symptoms: depression (12 mo) | MD = 0.2 (-0.2, 0.5) | - | - | No difference between the groups | 211 participants (1 RCT) | ⨁◯◯◯ VERY LOW ^6,7^ |
| Change of symptoms: anxiety (12 mo) | MD = 0.2 (-0.3, 0.5) | - | - | No difference between the groups^†^ | 211 participants (1 RCT) | ⨁◯◯◯ VERY LOW ^6,7^ |
| Change of symptoms: stress (12 mo) | MD = 0.2 (-0.2, 0.6) | - | - | No difference between the groups | 211 participants (1 RCT) | ⨁◯◯◯ VERY LOW ^6,7^ |
| Change of symptoms: quality of life (12 mo) | MD = 0.2 (-0.2, 0.6) | - | - | No difference between the groups^††^ | 211 participants (1 RCT) | ⨁◯◯◯ VERY LOW ^6,7^ |
| Problem-solving approach vs. UC |  |  |  |  |  |  |
| Change of symptoms: depression (18 mo) | - | - | - | No difference between the groups^†^ | 194 participants (1 RCT) | ⨁◯◯◯ VERY LOW ^2,8^ |
| Physical function (18 mo) | - | - | - | No difference between the groups^††^ | 194 participants (1 RCT) | ⨁◯◯◯ VERY LOW ^2,8^ |
| Cognitive function (18 mo) | - | - | - | No difference between the groups^††^ | 194 participants (1 RCT) | ⨁◯◯◯ VERY LOW ^2,8^ |
| Additional dialogue meeting vs. active treatment |  |  |  |  |  |  |
| Cognitive function | MD = –3.2 (-27.8, 21.3 | - | - | No difference between the groups | 60 participants (1 RCT) | ⨁◯◯◯ VERY LOW ^9,10^ |
| Adjuvant occupational therapy vs. UC |  |  |  |  |  |  |
| Change of symptoms: depression (18 mo) | Adjusted difference = -2.8 (-2.2, 1.8) |  |  | Improved symptoms of depression in the experimental group^††††^ | 117 participants (1 RCT) | ⨁◯◯◯ VERY LOW ^2,11^ |
| Physical function (18 mo) | Adjusted difference = |  |  |  |  |  |
| Self-efficacy (18 mo) | Adjusted difference = -0.2 (-2.2, 1.8) | - | - | No difference between the groups^†††^ | 117 participants (1 RCT) | ⨁◯◯◯ VERY LOW ^11,12^ |
| Stress treatment program vs. UC or waiting list |  |  |  |  |  |  |
| Change of symptoms: depression (3 mo) | Cohen’s *d* = 0.50 | - | - | No difference between the intervention group and UC. Waiting list control group showed significantly less improvement in depression | 199 participants (1 RCT) | ⨁◯◯◯ VERY LOW ^8,12^ |
| E-health module with collaborative occupational health care vs. UC |  |  |  |  |  |  |
| Change of symptoms: depression (12 mo) | - | - | - | No difference between the groups | 131 participants (1 RCT) | ⨁◯◯◯ VERY LOW ^6,12^ |

| **ANOVA**: Analysis of variance; **CI:** Confidence interval; **HR:** Hazard ratio; **HRQoL**: Health related quality of life; **MD**: mean difference; **Mo**: months; **MR**: multidisciplinary rehabilitation: **OR**: Odds ratio; **RCT**: Randomized controlled trial; **RR**: risk ratio; UC: usual care; **W-CBT**: Work-focused cognitive behavioral therapy ^†^ Hospital Anxiety and Depression Scale (HADS) ^††^ 36-Item Short Form Survey (SF-36) ^†††^ Expectations regarding work resumption ^††††^ Hamilton Rating Scale for Depression ^†††††^ Medical Outcomes Study-Short Form 1. Downgraded 2 levels for risk of bias (selection, attrition, and reporting bias) 2. Downgraded 1 level for imprecision (number of events) 3. Downgraded 1 level for imprecision (confidence interval encloses negative and positive effect) 4. Downgraded 2 levels for risk of bias (selection bias and performance bias) 5. Downgraded 2 levels for risk of bias (performance and reporting bias) 6. Downgraded 1 level for risk of bias (performance bias) 7. Downgraded 2 levels for imprecision (confidence interval encloses negative and positive effect) 8. Downgraded 2 levels for risk of bias (selection, performance, and attrition bias) 9. Downgraded 2 levels for risk of bias (selection, performance, attrition, and reporting bias) 10. Downgraded 1 level for imprecision (number of events and confidence interval encloses negative and positive effect) 11. Downgraded 2 levels for risk of bias (performance bias) 12. Downgraded 2 levels for imprecision (number of events and confidence interval encloses negative and positive effect) |
| --- |
